# The effects of testosterone on bone health in males with testosterone deficiency: a systematic review and meta-analysis

**DOI:** 10.1186/s12902-020-0509-6

**Published:** 2020-03-07

**Authors:** Zhichao Zhang, Deying Kang, Hongjun Li

**Affiliations:** 1 Andrology Center, Department of Urology, Peking University First Hospital; Institute of Urology, Peking University, No 8 Xishenku Street, Beijing, 100034 China; 2 Department of Evidence based Medicine and Clinical Epidemiology, West China Hospital, Sichuan University, No. 37 Guoxuexiang, Chengdu, 610041 China; 3 Urological Department of Peking Union Medical College Hospital (PUMCH), Peking Union Medical College, Chinese Academy of Medical Sciences, No. 1 Shuaifu, Eastern District, Beijing, 100730 China

**Keywords:** Testosterone, Aging, Males, Testosterone deficiency, Systematic review

## Abstract

**Background:**

Testosterone deficiency (TD) may induce a series of clinical symptoms.

Studies have shown that testosterone supplementation may prevent these unfavourable symptoms and improve patients’ quality of life. Given the conflicting findings across studies, this systematic review aims to evaluate the effects and risks associated with testosterone supplementation in middle-aged or aging males with TD.

**Methods:**

Electronic databases (MEDLINE, EMBASE, PubMed, and Cochrane.

Library were searched to December 2019. The risk of bias of individual included studies and the quality of the aggregate evidence were assessed using the GRADE approach. Our primary outcome was bone mineral density (BMD). Meta-analyses were performed. This systematic review was reported according to the PRISMA statement.

**Results:**

A total of 52 randomized controlled trials (RCTs) were included. When compared with placebo, testosterone supplementation did not increase total BMD (short-term: 1081 participants, MD − 0.01 g/cm^2^, 95% CI − 0.02 g/cm^2^ to 0.01 g/cm^2^; long-term: 156 participants, MD 0.04 g/cm^2^, 95% CI − 0.07 g/cm^2^ to 0.14 g/cm^2^), lumbar spine, hip, or femur neck BMD. Furthermore, testosterone supplementation did not decrease the risk of falling or fracture. Lastly, it was found that testosterone supplementation did not increase the risk of cardiovascular events (1374 participants, RR 1.28, 95% CI 0.62 to 2.64), all-cause mortality (729 participants, RR 0.55, 95% CI 0.29 to 1.04), or prostatic events. However, testosterone supplementation may improve sexual function and quality of life (1328 participants, MD -1.32, 95% CI − 2.11 to − 0.52).

**Conclusions:**

The effect of testosterone supplementation on BMD and the risk of falls or fracture remains inconclusive. However, supplementation may benefit patients in the areas of sexual function and quality of life without increasing the risk of cardiovascular events, all-cause mortality, or prostatic events. RCTs with a longer follow-up period are still required.

**Trial registration:**

We registered our protocol in PROSPERO (CRD42018109738).

## Background

Aging is associated with a 1% decline in testosterone levels in males, though the causes remain unclear [1]. Testosterone deficiency (TD) refers to a low level of serum testosterone and may induce a series of clinical symptoms [2]. Androgen deficiency may lead to dysfunctions of the skeletal, reproductive, and cardiovascular systems. Patients with TD also seem to be at higher risk of sustaining fractures^3.^ An epidemiological study [3] of 50,613 patients with prostate cancer who survived for at least five years reported a higher incidence of fractures in patients who received androgen-deprivation therapy (ADT) than in patients who did not (19.4% versus 12.6%, *p* < 0.001).

Given the association between TD and fracture revealed by the observational studies mentioned above, it is believed that androgen supplementation therapy can prevent osteoporosis and increase bone mass. However, several randomized controlled trials (RCTs) failed to demonstrate that testosterone supplementation increases bone density in patients with TD [4–6]. Furthermore, clinicians have also expressed concern about other associated risks of prescribing testosterone to middle–aged or aging patients with TD, especially the risk of cardiovascular and prostatic events [7–11]. Whether testosterone supplementation increases the risk of cardiovascular events remains a focus of debate. Two large cohort studies [9, 10] reported that testosterone therapy increases the risk of myocardial infarction. One RCT that enrolled 209 patients [11] also reported that the application of testosterone gel was associated with an increased risk of cardiovascular events. However, in another RCT [8], the authors found that the use of testosterone did not increase the risk of carotid artery intima-media thickness or coronary artery calcium in 308 men 60 years or older with low or low-normal testosterone levels.

There is also uncertainty among clinicians about whether testosterone supplementation in aging males is protective against other risks, such as all-cause mortality and prostate cancer. Although several systematic reviews [7, 12–15] on this topic have been published, they did not fully address the above questions [7, 12, 13]. While one review [13] investigated the effect of testosterone replacement on patients’ quality of life, it did not investigate the effect of testosterone replacement on bone mineral density (BMD), cardiovascular disease, and all-cause mortality. Three reviews [14–16] evaluated the efficacy of testosterone therapy in males with late-onset hypogonadism (LOH) and found that testosterone increased BMD. However, these reviews were either out of date or they omitted relevant studies; several RCTs reported no effect of testosterone on BMD after these reviews [17, 18] were published. Given these conflicting results, an update of the evidence regarding the impact of testosterone supplementation on BMD is required. Two systematic reviews investigated the risk of cardiovascular events after testosterone therapy, but the findings were inconsistent. One review [8] found that testosterone therapy increases the risk of cardiovascular events in aging males, while the other review [16] simply made reference to the controversy surrounding this issue. Given that the evidence to date is both conflicting and insufficient, this systematic review aims to evaluate the effect of testosterone supplementation on BMD and its potential risks (fracture, falling, all-cause mortality, cardiovascular disease, and prostate events) in middle-aged or aging males with TD.

## Methods

### Materials and methods

We registered our protocol in PROPERO (CRD42018109738). The systematic review and meta-analysis (study level) were conducted in alignment with the Cochrane Handbook of Interventional Reviews and reported in accordance with the PRISMA standard.

### Inclusion and exclusion criteria

Aging male adults (aged ≥40 years old) with a diagnosis of TD were included in this review. Because of the lack of a uniform definition of TD, we accepted any criteria used in the included studies to define TD. We only included studies involving patients with TD who were not interested in fertility and who were determined to have well-controlled obstructive sleep apnoea syndrome (OSAS). Any RCT in which testosterone therapy was used alone or in combination with other therapies (such as calcium or vitamin D) were included without restrictions regarding treatment dosage, frequency, and duration. Testosterone therapy might have included oral capsules, gels, patches, injections, pellets, sublingual testosterone. The comparator was placebo. The exclusion criteria were i) studies including patients with prostatic cancer who had received castration therapy (including endocrine therapy or testectomy) or androgen therapy; ii) studies including patients with testicular cancer; iii) studies including patients with primary hypogonadism induced by pituitary disease or pituitary surgery; iv) studies including patients with secondary hypogonadism (e.g., Paltauf’s dwarfism, pituitary tumour, acromegalia, or Cushing’s syndrome); and v) studies including patients who received other medications that influence androgen levels (e.g., finasteride, sildenafil).

Our primary outcome was total BMD. Secondary outcomes included lumbar spine BMD, total hip BMD, or other BMDs, the incidence rates of hip fracture, falling, total fracture, vertebral or non-vertebral fracture, all-cause mortality, and cardiovascular events (defined as myocardial infarction, angina, coronary artery disease, hypertension, stroke, or other definitions used in the original studies), as well as quality of life, total cost, sexual function, adverse events, prostate-specific antigen (PSA) level, and prostate events, such as prostate cancer or prostatitis.

### Searching and study screening

We conducted electronic searches in MEDLINE, Cochrane Library, EMBASE and PubMed on 9 December 2019. The search strategy was developed by an information specialist and is presented in Additional file 1. There was no limitation on language, document type, and publication status. We also hand searched the references of relevant systematic reviews to identify additional RCTs for inclusion. Two reviewers screened the search results. Disagreements were resolved by discussion with assistance from a third party if necessary.

### Data extraction and synthesis

Data from each study were extracted independently by two separate reviewers using a standardized data extraction form. Any disagreements were resolved by discussion with the assistance from a third party if necessary.

We synthesized data using a fixed-effect method for all analyses. An I^2^ estimate greater than or equal to 50% accompanied by a statistically significant χ^2^ statistic was interpreted as evidence of a substantial level of heterogeneity. Where substantial heterogeneity was found, we explored potential sources that may have caused this heterogeneity. If we could not definitively locate the sources of heterogeneity, we synthesized the data using a random-effects model. We summarised all dichotomous outcome data using risk ratios (RRs) and all continuous outcome data using mean differences (MDs) and calculated their respective 95% confidence intervals (CIs).

### Risk of bias assessment

We made the risk of bias judgments based on the methods endorsed by The Cochrane Collaboration, which included the following domains: patient allocation, blinding, selective reporting, attrition of study participants, and any other detected sources of bias [19].

### Additional analysis

We assessed the quality of the body of evidence for the primary and secondary outcomes based on the GRADE approach [20]. To test the robustness of the results of the synthesis, we conducted a trial sequential analysis (TSA) [21] for the primary outcomes. The required information size (RIS) was calculated based on the empirical mean difference and variance with a two-sided alpha of 0.05 and a beta of 0.20 [21].

## Results

### Study screening and characteristics of included studies

**Fig. 1:**
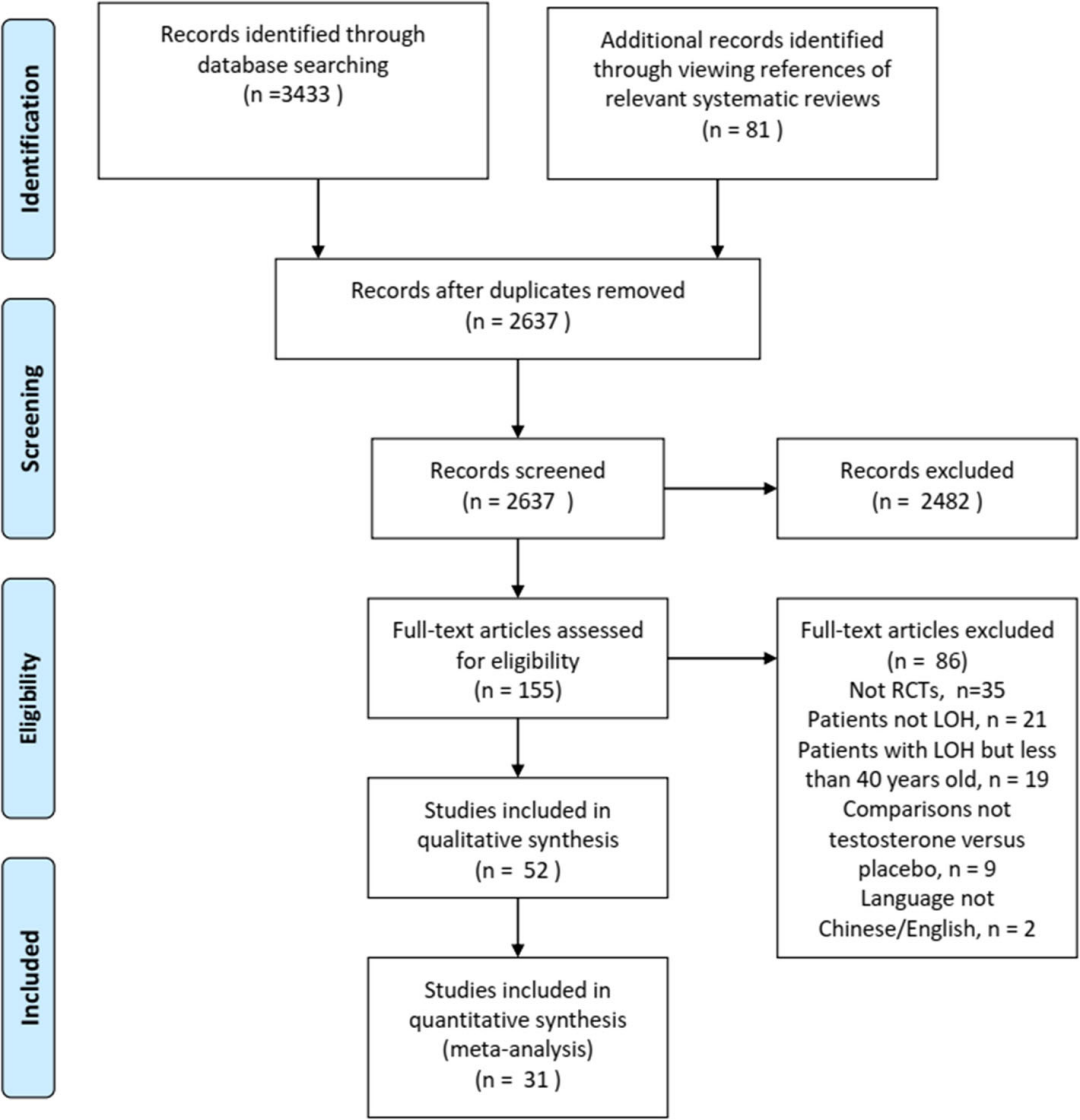
Study screening flow diagram, Notes: * 52 studies with 69 companion full-text articles

In total, 2637 references were screened, and 69 articles derived from 52 studies [4–6, 11, 17, 18, 22–67] were included after inspecting the full texts. The study screening process and the reasons for the exclusion of full texts are presented in Fig. 1. Fifty-two studies with 5067 participants met our study selection criteria. The study sample size ranged from 10 to 362. The average age of the participants ranged from 52 to 77.1 years, with BMIs ranging from 22.9 to 37.4. As reported in the included studies, there were 276 (5.45%) participants with CAD at baseline, 212 (4.18%) participants with dyslipidaemia or hyperlipidaemia, 261 (4.26%) participants with metabolic syndrome, 761 (15.02%) participants with diabetes mellitus, 419 (8.27%) participants with hypertension, 12 (0.24%) participants with prostatic disease, 186 (3.67%) participants with osteoporosis, 31 (0.61%) participants with osteoarthritis, and 57 (1.12%) participants with a history of fracture. Patient characteristics are summarized in Additional file 2.

### Risk of bias

**Fig. 2:**
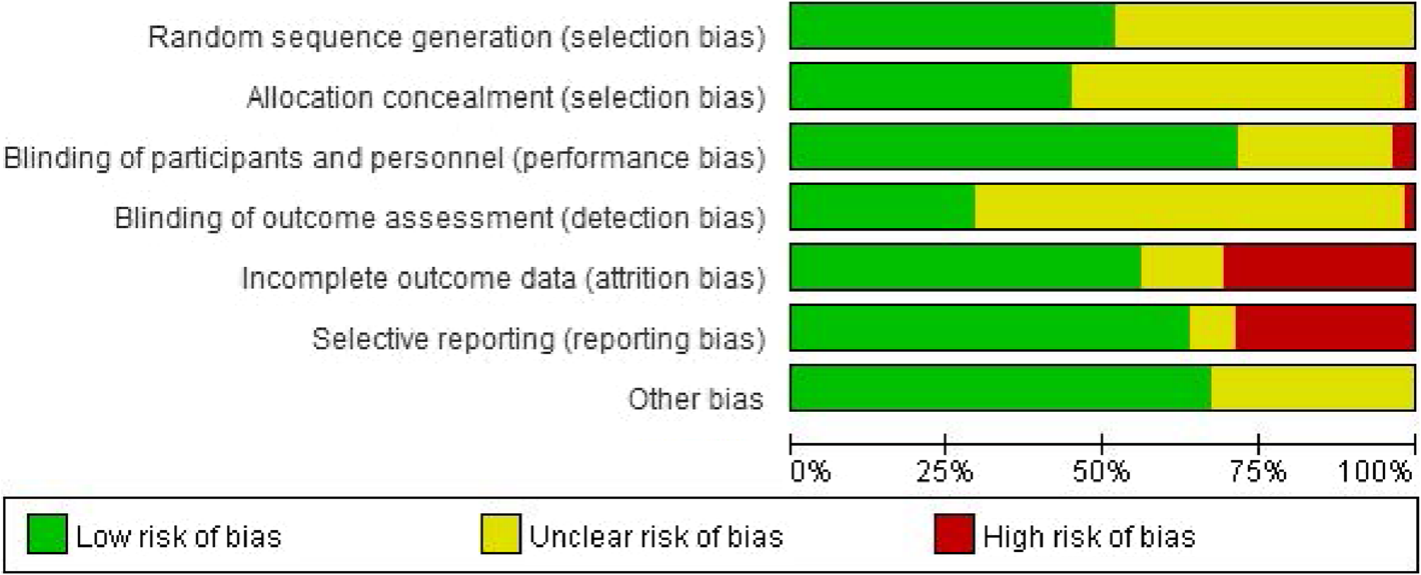
Risk of bias assessment

Figure 2 shows the overall results of the risk of bias of the included studies. The process of randomization was rated as low risk of bias in half of the included studies. With regard to blinding, 71% of the included studies stated that the participants and personnel were blinded to the treatment protocol. Twenty-nine studies were rated as low risk of bias in the domain of ‘incomplete outcome data’ because of the low attrition rate. Sixteen studies were rated as high risk of attrition bias, as the attrition rate was higher than 20% of the total sample size. Most studies (63%) were rated as low risk of bias in the ‘selective reporting domain’ because all measured outcomes were reported.

### Estimate of effect

Due to insufficient data, only a subgroup analysis of the treatment duration was conducted. We grouped all the included studies into short-term treatment duration (< 2 years) and long-term treatment duration (≥2 years).

### BMD

**Fig. 3:**
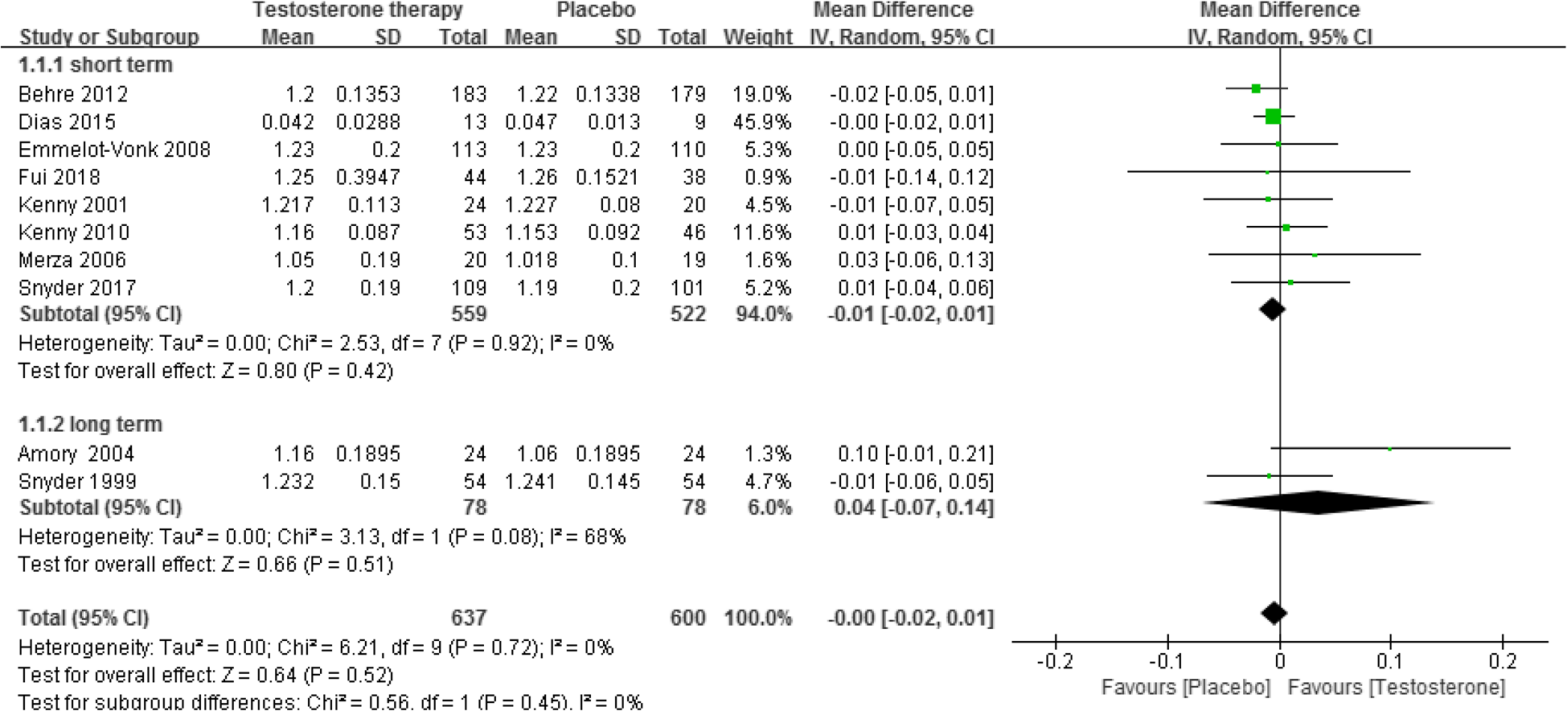
Testosterone versus placebo: Meta-analysis of total BMD

Ten RCTs [5, 6, 17, 18, 23, 36, 42, 48, 54, 68] reported this outcome. The results showed, when compared with placebo, testosterone supplementation did not increase total BMD in both the short-term (less than 2 years of treatment) (8 RCTs, 1081 participants, MD − 0.01 g/cm^2^, 95% CI − 0.02 g/cm^2^ to 0.01 g/cm^2^, low quality of evidence) and the long-term (more than 2 years of treatment) (2 RCTs, 156 participants, MD 0.04 g/cm^2^, 95% CI − 0.07 g/cm^2^ to 0.14 g/cm^2^, very low quality of evidence) (Fig. 3, Table 1). This outcome showed significant heterogeneity, but we were unable to identify the cause of heterogeneity. The optimal sample size for total BMD for the short- and long-term groups were 9310 and 1776, respectively (Additional file 3).

**Table 1:** Results of GRADE assessment

Outcomes	Outcomes Illustrative comparative risks* (95% CI)		Relative effect (95% CI)	No of Participants (studies)	Quality of the evidence (GRADE)	Comments
	Assumed risk	Corresponding risk				
	Placebo	Testosterone therapy alone				
**Total BMD (g/cm2) - average endpoint - short term** Follow-up: less than 24 months		The mean total bmd (g/cm2) - average endpoint - short term in the intervention groups was **0.01 lower** (0.02 lower to 0.01 higher)		1081 (8 studies)	⊕ ⊕ ⊝⊝ **low**^1,2^	
**Total BMD (g/cm2) - average endpoint - long term** Follow-up: more than 24 months		The mean total bmd (g/cm2) - average endpoint - long term in the intervention groups was **0.04 higher** (0.07 lower to 0.14 higher)		156 (2 studies)	⊕⊝⊝⊝ **very low**^1,2,3,4^	
**Fracture - short term** Follow-up: 12 months	**Study population**		**RR 0.92** (0.31 to 2.76)	211 (1 study)	⊕ ⊕ ⊝⊝ **low**^4,5^	
	**59 per 1000**	**55 per 1000** (18 to 164)				
	**Moderate**					
	**59 per 1000**	**54 per 1000** (18 to 163)				
**Falls - short term** Follow-up: 6 months	**Study population**		**RR 0.70** (0.34 to 1.45)	262 (1 study)	⊕ ⊕ ⊝⊝ **low**^4,5^	
	**121 per 1000**	**85 per 1000** (41 to 176)				
	**Moderate**					
	**121 per 1000**	**85 per 1000** (41 to 175)				
**Mortality - short term** Follow-up: less than 24 months	**Study population**		**RR 0.62** (0.3 to 1.31)	598 (4 studies)	⊕ ⊕ ⊝⊝ **low**^3,5^	
	**50 per 1000**	**31 per 1000** (15 to 65)				
	**Moderate**					
	**13 per 1000**	**8 per 1000** (4 to 17)				
**Mortality - long term** Follow-up: more than 24 months	**Study population**		**RR 0.39** (0.1 to 1.42)	131 (1 study)	⊕⊝⊝⊝ **very low**^3,4,5^	
	**113 per 1000**	**44 per 1000** (11 to 160)				
	**Moderate**					
	**113 per 1000**	**44 per 1000** (11 to 160)				
**Cardiovascular event - short term** Follow-up: less than 24 months	**Study population**		**RR 1.2** (0.44 to 3.26)	1204 (8 studies)	⊕⊝⊝⊝ **very low**^1,3,5^	
	**50 per 1000**	**60 per 1000** (22 to 164)				
	**Moderate**					
	**39 per 1000**	**47 per 1000**(17 to 127)				
**Cardiovascular event - long term** Follow-up: more than 24 months	**Study population**		**RR 1.42** (0.66 to 3.05)	170 (2 studies)	⊕ ⊕ ⊝⊝ **low**^4,5^	
	**116 per 1000**	**165 per 1000** (77 to 355)				
	**Moderate**					
	**124 per 1000**	**176 per 1000**(82 to 378)				
**Quality of life - average endpoint (AMS, high = worse) - short term** Follow-up: less than 24 months		The mean quality of life - average endpoint (ams, high = worse) - short term in the intervention groups was **1.32 lower** (2.11 to 0.52 lower)		1328 (8 studies)	⊕ ⊕ ⊕⊝ **moderate**^6^	
**Sexual function - average endpoint (IIEF score, high = well) - short term** Follow-up: less than 24 years		The mean sexual function - average endpoint (iief score, high = well) - short term in the intervention groups was **1.48 higher** (0.05 to 2.91 higher)		503 (4 studies)	⊕ ⊕ ⊝⊝ **low**^1,3,7^	

(To be inserted in Results at Line 204 Page 9)

**Fig. 4:**
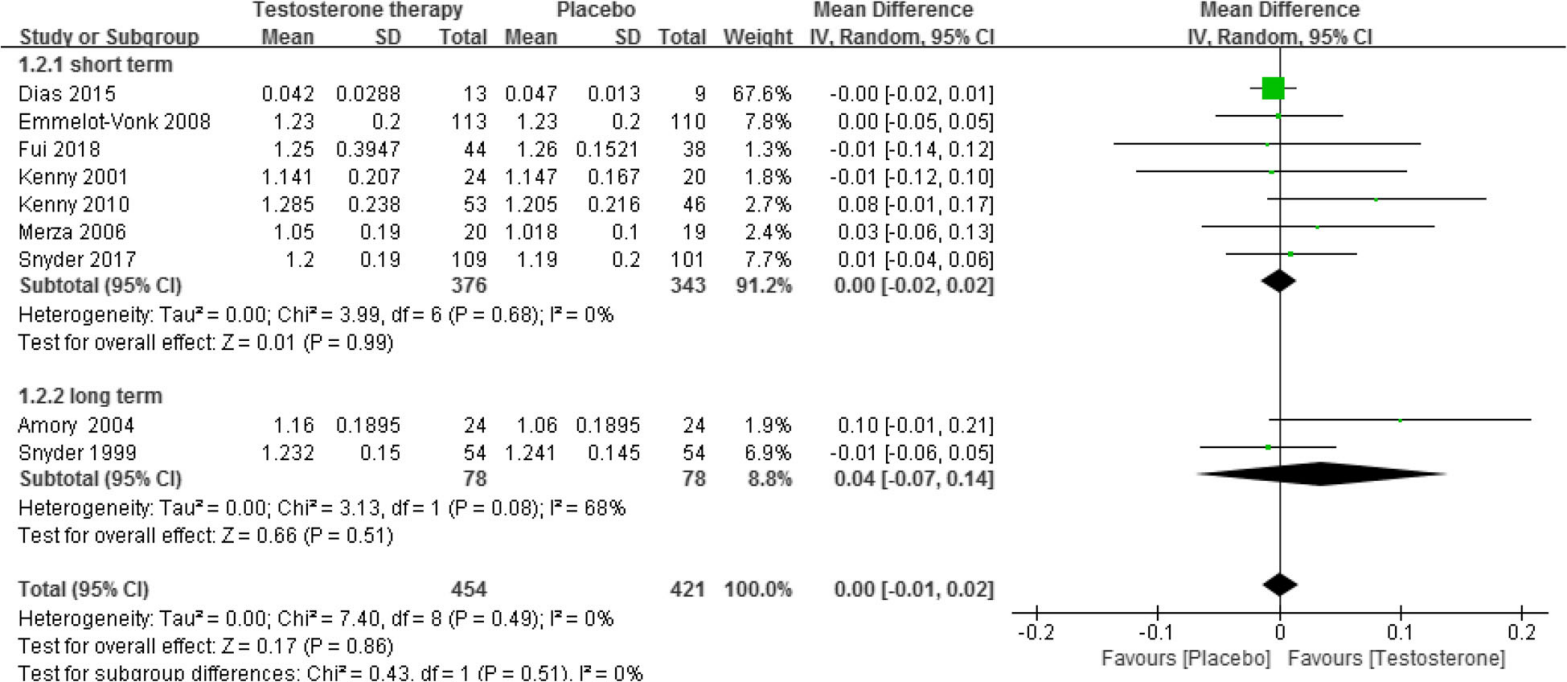
Testosterone versus placebo: Meta-analysis of lumbar spine BMD

**Fig. 5:**
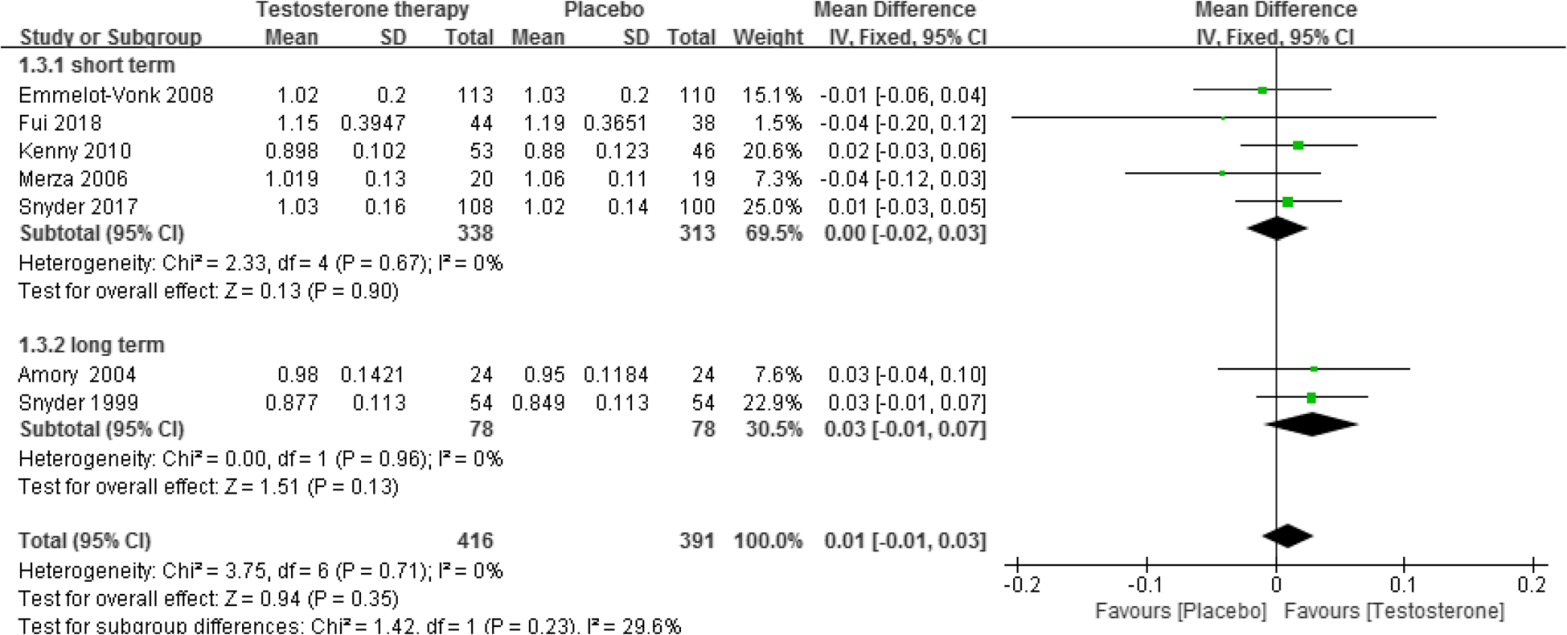
Testosterone versus placebo: Meta-analysis of hip BMD

**Fig. 6:**
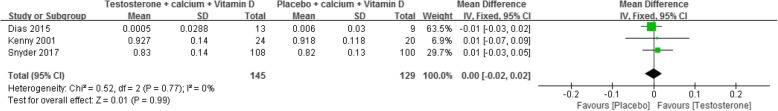
Testosterone versus placebo: Meta-analysis of femur neck BMD

This nonsignificant effect was also observed for lumbar spine BMD (short-term: 7 RCTs [6, 17, 18, 36, 42, 48, 68], 719 participants, MD 0.00 g/cm^2^, 95% CI − 0.02 g/cm^2^ to 0.02 g/cm^2^; long-term: 2 RCTs [23, 54], 156 participants, MD 0.04 g/cm^2^, 95% CI − 0.07 g/cm^2^ to 0.14 g/cm^2^, Fig. 4), hip BMD (short-term: 5 RCTs [17, 18, 36, 42, 48], 651 participants, MD 0.00 g/cm^2^, 95% CI − 0.02 g/cm^2^ to 0.03 g/cm^2^; long-term: 2 RCTs [23, 54], 156 participants, MD 0.03 g/cm^2^, 95% CI − 0.01 g/cm^2^ to 0.07 g/cm^2^, Fig. 5) and femur neck BMD (short-term: 3 RCT s [6, 18, 68], 274 participants, MD 0.00 g/cm^2^, 95% CI − 0.02 g/cm^2^ to 0.02 g/cm^2^, Fig. 6).

### Incidence rate of fracture

One RCT [18] reported the incidence rate of fracture. No significant difference was found between the groups (short-term, 211 participants, RR 0.92, 95% CI 0.31 to 2.76; low quality of evidence, Table 1). No study reported the incidence rate of fracture in specific bone sites, such as the hip or vertebrae.

### Incidence rate of falling

One RCT [56] reported the incidence rate of falling. The results showed no significant difference between the groups (short-term, 262 participants, RR 0.70, 95% CI 0.34 to 1.45; low quality of evidence, Table 1).

### All-cause mortality

**Fig. 7:**
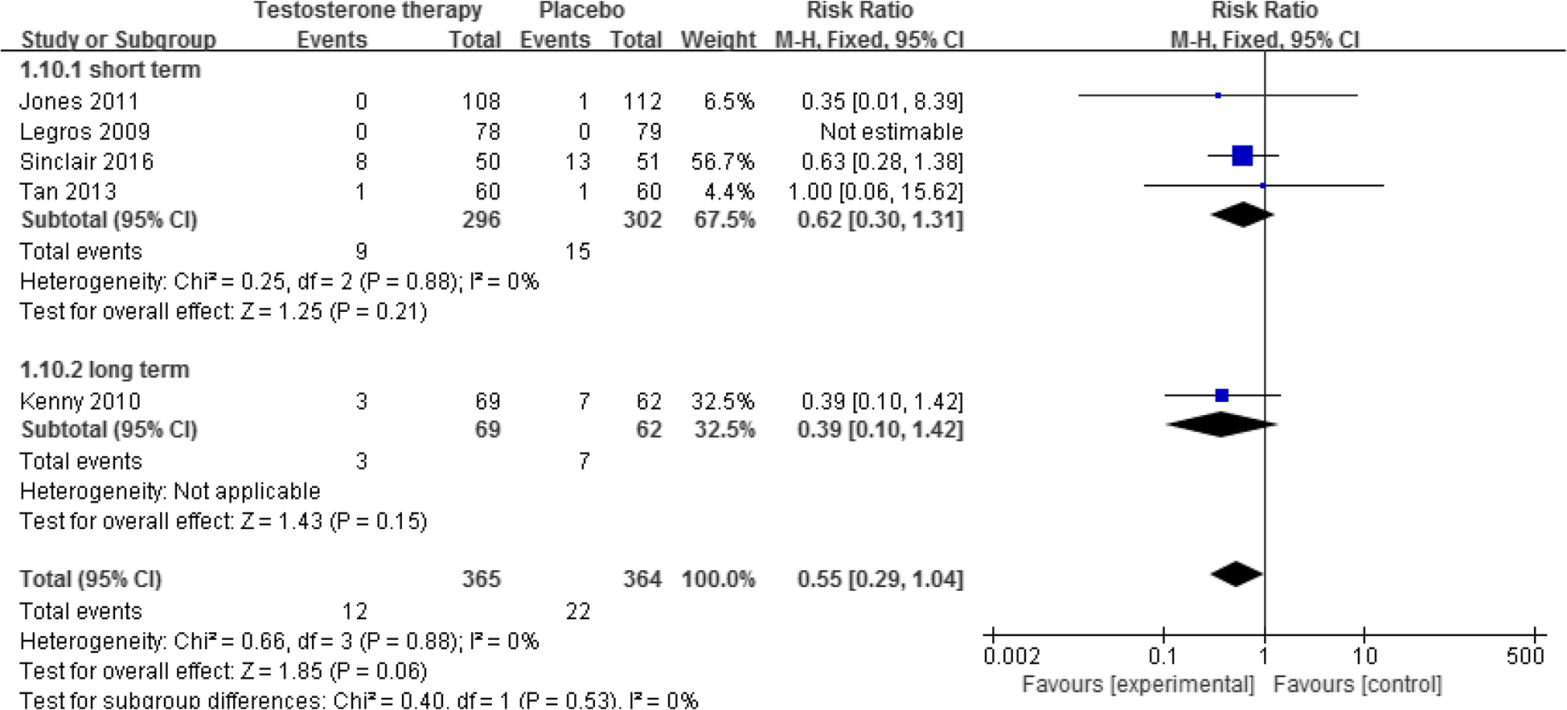
Testosterone versus placebo: Meta-analysis of all-cause mortality

Five RCTs [41–43, 59, 67] reported this outcome. The results showed that when compared with placebo, testosterone supplementation decreased the risk of all-cause mortality, however, the difference was not statistically significant (5 RCTs [41–43, 59, 67], 729 participants, RR 0.55, 95% CI 0.29 to 1.04; Fig. 7), either in the short-term (low quality of evidence, Table 1) and the long-term (Fig. 7; very low quality of evidence, Table 1).

### Incidence of cardiovascular event

**Fig. 8:**
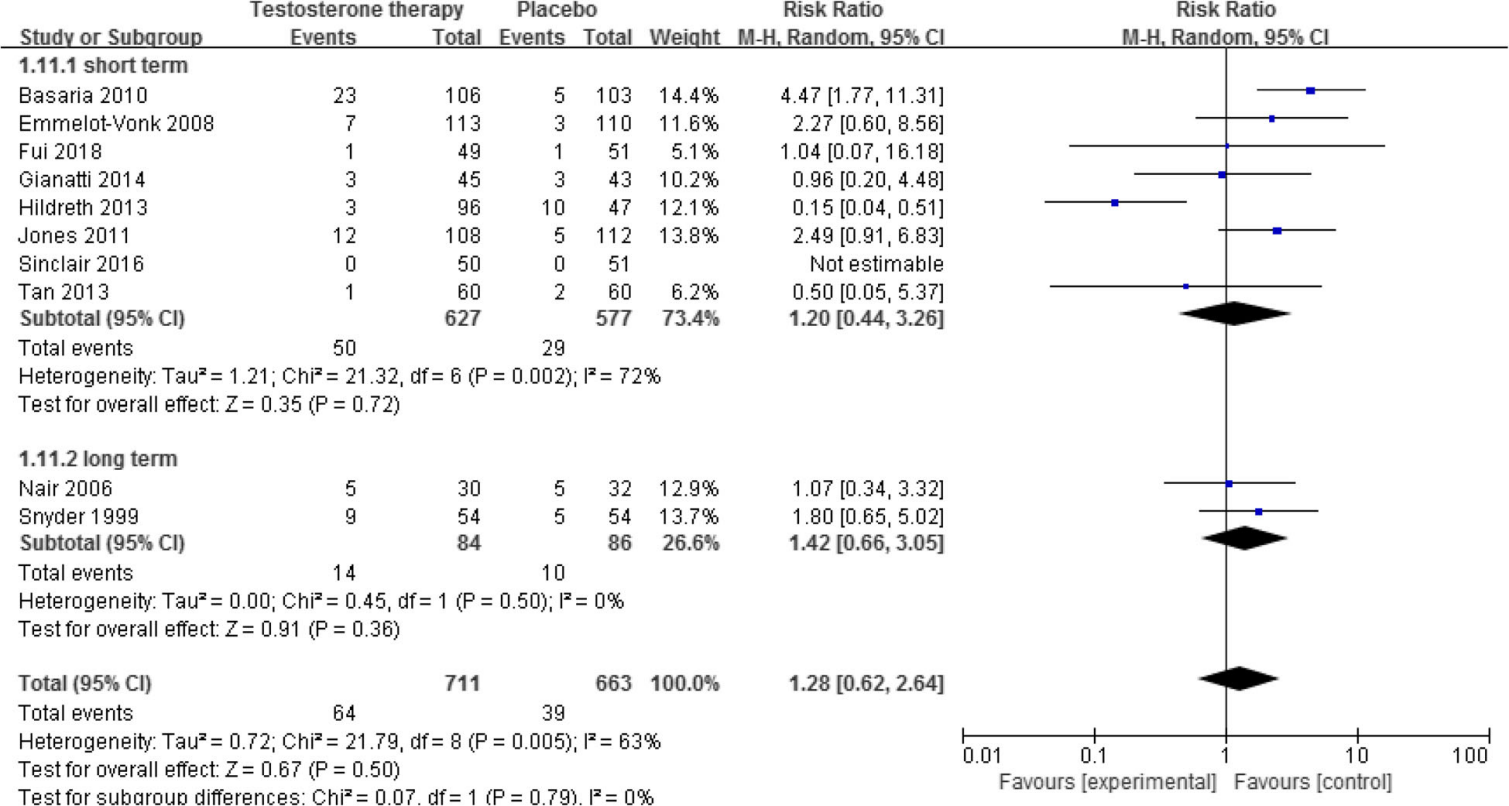
Testosterone versus placebo: Meta-analysis of risk of cardiovascular event

Ten RCTs with 12 references [11, 17, 36, 40, 41, 49, 54, 59, 66, 67, 69, 70] reported this outcome. The results showed no significant difference in the risk of cardiovascular events between the placebo and testosterone supplementation groups (1374 participants, RR 1.28, 95% CI 0.62 to 2.64). This outcome showed significant heterogeneity (I^2^ = 72%), which was induced by one study [40]. However, after comparing variables, such as patient characteristics, different types of testosterone, and treatment duration between this and the other studies, we failed to identify the specific source of heterogeneity. There was no significant difference between groups both in the short- and long-term subgroup analysis (Fig. 8; low quality of evidence, Table 1).

### Quality of life

Eight RCTs [5, 39, 41, 43, 50, 56, 59, 63] measured this outcome using the Aging Males’ Symptoms (AMS) scale. The results demonstrated that testosterone supplementation improved patients’ quality of life in the short-term (1328 participants, MD -1.32, 95% CI − 2.11 to − 0.52, Additional file 4; moderate quality of evidence, Table 1).

### Sexual function

Four RCTs [39, 41, 59, 65] measured this outcome by using the International Index of Erectile Function-5 (IIEF-5) scale. The results demonstrated that testosterone supplementation improved sexual function in the short-term (503 participants, MD 1.48, 95% CI 0.05 to 2.91, Additional file 5). This outcome had a significant level of heterogeneity. After exploring the sources, we found that Tan 2013 [54] was an outlier, but we could not identify the specific causes for the heterogeneity. The quality of evidence was low (Table 1).

### Adverse events

Eight RCTs with nine references [5, 32, 36, 43, 55, 56, 59, 66, 69] reported the total number of adverse events between the testosterone supplementation and placebo groups. No significant difference between the groups was found in each study, except for Tan 2013 who reported a lower incidence of adverse events in the testosterone group. Due to the heterogeneity found in the total adverse events across studies, we did not pool the data in the meta-analysis (Additional file 6).

### PSA level

Fifteen RCTs [4, 5, 23, 31, 33, 36, 42, 46, 50, 52, 54, 56, 59, 60, 71] showed a slightly higher serum PSA level in the testosterone supplementation group (1514 participants, MD 0.15, 95% CI 0.04 to 0.27, Additional file 7). This difference was observed in the short- but not the long-term (Additional file 7). Six RCTs also reported the risk of PSA among the groups, but no significant difference was found between the groups (1090 participants, RR 1.14, 95% CI 0.71 to 1.81, Additional file 8).

### Prostate events

One study [54] reported the incidence rate of prostate events in the placebo and testosterone supplementation groups. The prostate events included prostatitis, prostate nodule, prostate cancer, and PSA increase. No significant difference was found between the groups (1 RCT, 108 participants, RR 1.45, 95% CI 0.75 to 2.84).

## Discussion

This review included 5067 participants with TD. Evidence showed that compared with placebo, testosterone supplementation did not i) increase total BMD, vertebral, hip and femoral BMD; ii) decrease the risk of falling or fracture; or iii) increase the risk of cardiovascular events, all-cause mortality or prostatic events, such as PSA increase or prostatitis; however, testosterone supplementation was associated with improved quality of life and sexual function. Nonetheless, the above findings may be influenced by the presence of attrition bias and selective reporting in individual RCTs. Furthermore, the small total sample size and the unexplained heterogeneity between studies also impacted the quality of the body of evidence, especially for long-term outcomes and the risk of cardiovascular events. In terms of sexual function and quality of life, the indirect approach used to interpret the results of the screening tools somewhat reduces our level of confidence in these findings. All the included studies used surrogate outcome measurements, namely the mean difference in the scores of each scale, to reflect improvement in these two outcomes. However, clinicians must also consider whether the differences in the scores between the two compared groups are clinically significant.

Testosterone receptors are widely distributed in bone tissues. When combined with these receptors, testosterone facilitates skeletal growth and development, for instance by stimulating the proliferation of preosteoblasts and the differentiation of osteoblasts (non-dependent oestrogen conversion) and by promoting the maturation and ossification of cartilage cells and deposits of calcium on bone [72]. Theoretically, testosterone supplementation can improve bone health in patients with TD. However, the current meta-analysis failed to demonstrate this effect, a finding that is consistent with previous systematic reviews [15, 73–75]. Contrary to our findings, a guideline published in 2010 [76] stated that although testosterone had no effect on vertebral, hip and femoral BMD, it was associated with an increase in lumbar BMD. A possible reason for this inconsistent finding is that this guideline focused on patients with osteoporosis, while we included only a very small proportion of participants with osteoporosis. Nonetheless, even with our negative finding, there are several reasons why caution must be exercised in concluding that testosterone does not affect BMD. First, the finding that testosterone supplementation did not improve BMD in the short-term (< 2 years) may due to inadequate duration of treatment. It is well known that the effect of testosterone on BMD is only evident after more than 2 years of use. However, only 156 participants from two studies used testosterone for > 2 years, and the sample size is too small to detect a significant difference between the groups. Second, although all participants were androgen deficient, most did not have any abnormality in bone mass density or any evidence of osteoporosis at baseline; therefore, the change in BMD before and after testosterone supplementation may be nonsignificant. We also did not find any difference in the risk of fall or fracture between the testosterone supplementation and the placebo groups, though this may also be due to inadequate treatment duration and the small sample size.

Several studies [7, 9, 11] indicated that testosterone increases the risk of cardiovascular events. However, we did not find this effect in our meta-analysis, possibly because only a small proportion (5.45%) of participants in our review had a history of CAD at baseline, while several studies [9–11] included a larger number of patients with a history of CAD. Furthermore, the age range of participants also differs between our review and the above studies, with the latter including participants older than 60 years compared with our inclusion of participants over 40 years of age. One cohort study [10] indicated that a history of CAD and an age greater than 65 years were risk factors for cardiovascular events in patients treated with testosterone.

With regard to all-cause mortality, our review found that testosterone did not decrease the risk of all-cause mortality in patients with TD. We concluded that this negative result was due to inadequate sample size, as there was an obvious trend towards a reduction in the rate of all-cause mortality in the testosterone supplementation group; however, the 95% confidence interval was too wide to detect a significant difference. While this result is consistent with another review [72] in which testosterone supplementation was found not to increase the incidence of severe adverse events, including mortality, it is contrary to the finding reported in a cohort study of a positive association between testosterone supplementation and all-cause mortality [10]. These inconsistent findings may in part be explained by the variations in the baseline characteristics of the participants, especially the differences in ages and associated comorbidities. More RCTs are clearly needed to better identify the effect of testosterone supplementation on mortality.

Three studies [12, 13, 77] found that testosterone therapy increases sexual function in patients with a low testosterone level, which is consistent with our finding. We also found improved quality of life in the testosterone therapy group. However, one study did not find this favourable effect [76]. In that study [76], the included participants had complex comorbidities, which may explain this difference. In our meta-analysis fewer participants had comorbidities, and thus, a significant improvement in quality of life may have been more evident.

Persistent concerns revolve around whether testosterone supplementation increases the risk of prostate cancer or BPH. Consistent with other observational studies [78–80], this review did not find an association between testosterone supplementation and prostate cancer. Interestingly, subnormal testosterone levels have been reported to be associated with high-grade prostate cancer [81].

This systematic review has some strengths. First, the search strategy was developed by a professional information specialist. In addition, we searched both electronic databases and hand searched the references of relevant systematic reviews. This approach allowed us to collect as many relevant RCTs as possible. Second, the study screening and data extraction process were conducted by two researchers independently to minimize bias.

The systematic review also has some limitations. For instance, the long-term data for primary or secondary outcomes were insufficient to detect a clear difference between the groups. Furthermore, significant heterogeneity between populations was identified, such as the definition of TD or LOH and differences in the presence of comorbidities at baseline. Despite the presence of significant heterogeneity, we were unable to determine whether the variations in the effect of testosterone supplementation across subgroup populations were due to insufficient data.

## Conclusion

### Implications for practice

The effect of testosterone supplementation on BMD and on the risk of falling or fracture in patients with TD remains inconclusive. However, testosterone supplementation may improve sexual function and quality of life without increasing the risk of CAD, all-cause mortality, and prostate diseases.

### Implications for research

Further research is needed with RCTs that adequately report methods used for generating random allocation sequences. Larger RCTs with long-term data on the effect of testosterone supplementation on BMD, risk of fracture or falling, CAD, and all-cause mortality are required. Lastly, RCTs are also needed that focus on our predefined subgroup population of TD patients, such as those between 40 and 65 years of age as well as patients with osteoporosis or a history of CAD.

## Supplementary information

**Additional file 1.** Search Strategies.

**Additional file 2.** Characteristics of Participants.

**Additional file 3.** Trial Sequential Analysis.

**Additional file 4.** Testosterone versus placebo: Meta-analysis of quality of life (AMS scale).

**Additional file 5.** Testosterone versus placebo: Meta-analysis of sexual function (IIEF-5 scale).

**Additional file 6.** Testosterone versus placebo: Forest plot of total adverse events.

**Additional file 7.** Testosterone versus placebo: Meta-analysis of serum PSA level.

**Additional file 8.** Testosterone versus placebo: Meta-analysis of risk of PSA increase.

**Additional file 9.**

**Additional file 10.**

## Data Availability

The datasets used and/or analysed during the current study are available from the corresponding author upon reasonable request.

## Abbreviations

ADT: Androgen-deprivation therapy
AMS: Aging Males’ Symptoms
BMD: Bone mineral density
CAD: Cardiovascular disease
CIs: Confidence intervals
IIEF-5: International Index of Erectile Function-5
MDs: Mean differences
PSA: Prostate specific antigen
RCT: Randomized controlled trial
RIS: Required information size
RR: Risk ratio
TD: Testosterone deficiency
TSA: Trial sequential analysis
