# Supplementary material for: The effects of testosterone on bone health in males with testosterone deficiency: a systematic review and meta-analysis
Source: BMC Endocr Disord. 2020 Mar 7;20:33. doi: 10.1186/s12902-020-0509-6 (PMC7060639; doi:10.1186/s12902-020-0509-6)
Supplement: Supplementary file 1 — Additional file 1. Search Strategies. [file 12902_2020_509_MOESM1_ESM.docx]

**Additional file 1: Search Strategies**

***A. Cochrane Library***

#1 MeSH descriptor: [Osteoporosis] this term only

#2 osteop*:ti,ab,kw (Word variations have been searched)

#3 MeSH descriptor: [Bone Density] this term only

#4 (bone near/2 densit*):ti,ab,kw (Word variations have been searched)

#5 (bone near/2 loss):ti,ab,kw (Word variations have been searched)

#6 (bone* next fragil*):ti,ab,kw (Word variations have been searched)

#7 ("bone mineral" next content*):ti,ab,kw (Word variations have been searched)

#8 (bmd or bmc):ti,ab,kw (Word variations have been searched)

#9 MeSH descriptor: [Fractures, Bone] explode all trees

#10 fractur*:ti,ab,kw (Word variations have been searched)

#11 #1 or #2 or #3 or #4 or #5 or #6 or #7 or #8 or #9 or #10

#12 MeSH descriptor: [Testosterone] explode all trees

#13 (testosterone or "8 isotestosterone" or "8-isotestosterone" or adrotest or Andriol or "andro 100" or androderm or androfort or androgel or androlin or andronaq or andropatch or androsorb or androstenolone or "Androtardyl 250" or androtest or androtop or andrusol or aquaviron or Axiron or "beta testosterone" or "bio t gel" or "col 1621" or col1621 or Delatestryl or "depot hormon-m" or Fortesa or fortigel or "geno cristaux" or histerone or homosteron or hydroxyandrostenone or Intrinsa or libigel or livensa or mertestate or natesto or Nebido or neotestis or "nsc 9700" or nsc9700 or opterone or "oreton f" or orquisteron or "Pantestone 40" or "percutacrine androgenique" or "percutacrine androgine" or primotest* or "Restandol Testocaps" or sterotate or Striant or sustanon or sustenon or synandrol or teslen or testamone or testandrone or testaqua or testerone or testim or Testim or "testo enant" or testoderm or testogel or "testoject 50" or testolin or testoluton or testopel or testosterone? or testro or testrone or testryl or Tostran or tostrelle or tostrex or Virormone or virosterone or vogelxo):ti,ab,kw (Word variations have been searched)

#14 MeSH descriptor: [Hypogonadism] explode all trees

#15 hypogonadism:ti,ab,kw (Word variations have been searched)

#16 #12 or #13 or #14 or #15

#17 #11 and #16 in Trials

***B. EMBASE***

1 exp osteoporosis/

2 osteop*.tw,kw.

3 bone density/

4 (bone adj2 densit*).tw,kw.

5 (bone adj2 loss).tw,kw.

6 (bone* adj fragil*).tw,kw.

7 bone mineral content*.tw,kw.

8 (bmd or bmc).tw,kw.

9 exp fracture/

10 fractur*.tw,kw.

11 or/1-10

12 exp testosterone/

13 (testosterone or "8 isotestosterone" or 8-isotestosterone or adrotest or Andriol or "andro 100" or androderm or androfort or androgel or androlin or andronaq or andropatch or androsorb or androstenolone or "Androtardyl 250" or androtest or androtop or andrusol or aquaviron or Axiron or beta testosterone or "bio t gel" or col 1621 or col1621 or Delatestryl or "depot hormon-m" or Fortesa or fortigel or "geno cristaux" or histerone or homosteron or hydroxyandrostenone or Intrinsa or libigel or livensa or mertestate or natesto or Nebido or neotestis or "nsc 9700" or nsc9700 or opterone or "oreton f" or orquisteron or "Pantestone 40" or "percutacrine androgenique" or "percutacrine androgine" or primotest* or "Restandol Testocaps" or sterotate or Striant or sustanon or sustenon or synandrol or teslen or testamone or testandrone or testaqua or testerone or testim or Testim or "testo enant" or testoderm or testogel or "testoject 50" or testolin or testoluton or testopel or testosterone? or testro or testrone or testryl or Tostran or tostrelle or tostrex or Virormone or virosterone or vogelxo).mp.

14 hypogonadism/

15 hypogonadism.tw,kw.

16 or/12-15

17 11 and 16

18 Female/ not Male/

19 17 not 18

20 (clin$ adj2 trial).mp.

21 ((singl$ or doubl$ or trebl$ or tripl$) adj (blind$ or mask$)).mp.

22 (random$ adj5 (assign$ or allocat$)).mp.

23 randomi$.mp.

24 crossover.mp.

25 exp randomized-controlled-trial/

26 exp double-blind-procedure/

27 exp crossover-procedure/

28 exp single-blind-procedure/

29 exp randomization/

30 or/20-29

31 19 and 30

***C. MEDLINE***

1 Osteoporosis/

2 osteop*.tw,kw,kf.

3 Bone Density/

4 (bone adj2 densit*).tw,kw,kf.

5 (bone adj2 loss).tw,kw,kf.

6 (bone* adj fragil*).tw,kw,kf.

7 bone mineral content*.tw,kw,kf.

8 (bmd or bmc).tw,kw,kf.

9 exp Fractures, Bone/

10 fractur*.tw,kw,kf.

11 or/1-10

12 exp Testosterone/

13 (testosterone or "8 isotestosterone" or 8-isotestosterone or adrotest or Andriol or "andro 100" or androderm or androfort or androgel or androlin or andronaq or andropatch or androsorb or androstenolone or "Androtardyl 250" or androtest or androtop or andrusol or aquaviron or Axiron or beta testosterone or "bio t gel" or col 1621 or col1621 or Delatestryl or "depot hormon-m" or Fortesa or fortigel or "geno cristaux" or histerone or homosteron or hydroxyandrostenone or Intrinsa or libigel or livensa or mertestate or natesto or Nebido or neotestis or "nsc 9700" or nsc9700 or opterone or "oreton f" or orquisteron or "Pantestone 40" or "percutacrine androgenique" or "percutacrine androgine" or primotest* or "Restandol Testocaps" or sterotate or Striant or sustanon or sustenon or synandrol or teslen or testamone or testandrone or testaqua or testerone or testim or Testim or "testo enant" or testoderm or testogel or "testoject 50" or testolin or testoluton or testopel or testosterone? or testro or testrone or testryl or Tostran or tostrelle or tostrex or Virormone or virosterone or vogelxo).mp.

14 HYPOGONADISM/

15 hypogonadism.tw,kw,kf.

16 or/12-15

17 11 and 16

18 Females/ not Male/

19 17 not 18

20 exp clinical trial/

21 exp randomized controlled trials/

22 exp double-blind method/

23 exp single-blind method/

24 exp cross-over studies/

25 randomized controlled trial.pt.

26 clinical trial.pt.

27 controlled clinical trial.pt.

28 (clinic$ adj2 trial).mp.

29 (random$ adj5 control$ adj5 trial$).mp.

30 (crossover or cross-over).mp.

31 ((singl$ or double$ or trebl$ or tripl$) adj (blind$ or mask$)).mp.

32 randomi$.mp.

33 (random$ adj5 (assign$ or allocat$ or assort$ or reciev$)).mp.

34 or/20-33

35 19 and 34

***D. PUBMED***

(((("Osteoporosis"[Mesh:noexp]) OR (osteop*[Text Word]) OR ("Bone Density"[Mesh:noexp]) OR (bone densit*[Text Word]) OR (bone loss[Text Word]) OR (bone* AND fragil*[Text Word]) OR (bone mineral content*[Text Word]) OR (bmd[Text Word] OR bmc[Text Word]) OR ("Fractures, Bone"[Mesh]) OR (fractur*[Text Word])) AND and AND (("Testosterone"[Mesh]) OR (testosterone*[Text Word]) OR ("8 isotestosterone"[Text Word] OR "8-isotestosterone"[Text Word] OR adrotest[Text Word] OR Andriol[Text Word] OR "andro 100"[Text Word] OR androderm[Text Word] OR androfort[Text Word] OR androgel[Text Word] OR androlin[Text Word] OR andronaq[Text Word] OR andropatch[Text Word] OR androsorb[Text Word] OR androstenolone[Text Word] OR "Androtardyl 250"[Text Word] OR androtest[Text Word] OR androtop[Text Word] OR andrusol[Text Word] OR aquaviron[Text Word] OR Axiron[Text Word] OR "beta testosterone"[Text Word] OR "bio t gel"[Text Word] OR "col 1621"[Text Word] OR col1621[Text Word] OR Delatestryl[Text Word] OR "depot hormon-m"[Text Word] OR Fortesa[Text Word] OR fortigel[Text Word] OR "geno cristaux"[Text Word] OR histerone[Text Word] OR homosteron[Text Word] OR hydroxyandrostenone[Text Word] OR Intrinsa[Text Word] OR libigel[Text Word] OR livensa[Text Word] OR mertestate[Text Word] OR natesto[Text Word] OR Nebido[Text Word] OR neotestis[Text Word] OR "nsc 9700"[Text Word] OR nsc9700[Text Word] OR opterone[Text Word] OR "oreton f"[Text Word] OR orquisteron[Text Word] OR "Pantestone 40"[Text Word] OR "percutacrine androgenique"[Text Word] OR "percutacrine androgine"[Text Word] OR primotest*[Text Word] OR "Restandol Testocaps"[Text Word] OR sterotate[Text Word] OR Striant[Text Word] OR sustanon[Text Word] OR sustenon[Text Word] OR synandrol[Text Word] OR teslen[Text Word] OR testamone[Text Word] OR testandrone[Text Word] OR testaqua[Text Word] OR testerone[Text Word] OR testim[Text Word] OR Testim[Text Word] OR "testo enant"[Text Word] OR testoderm[Text Word] OR testogel[Text Word] OR "testoject 50"[Text Word] OR testolin[Text Word] OR testoluton[Text Word] OR testopel[Text Word] OR testro[Text Word] OR testrone[Text Word] OR testryl[Text Word] OR Tostran[Text Word] OR tostrelle[Text Word] OR tostrex[Text Word] OR Virormone[Text Word] OR virosterone[Text Word] OR vogelxo[Text Word]) OR ("Hypogonadism"[Mesh:noexp]) OR (hypogonadism[Text Word]))) NOT (("Female"[Mesh]) NOT "Male"[Mesh])) AND and AND (((randomized controlled trial[pt]) OR (controlled clinical trial[pt]) OR (randomized[tiab]) OR (placebo[tiab]) OR (drug therapy[sh]) OR (randomly[tiab]) OR (trial[tiab]) OR (groups[tiab])) NOT (animals[mh] NOT humans[mh]))
