# Supplementary material for: The effects of testosterone on bone health in males with testosterone deficiency: a systematic review and meta-analysis
Source: BMC Endocr Disord. 2020 Mar 7;20:33. doi: 10.1186/s12902-020-0509-6 (PMC7060639; doi:10.1186/s12902-020-0509-6)
Supplement: Supplementary file 2 — Additional file 2. Characteristics of Participants. [file 12902_2020_509_MOESM2_ESM.docx]

Additional file 2:

Table 1 Characteristics of participants

| **STUDY ID** | **SAMPLE** | **COUNTRY** | **DIAGNOSIS** | **DIAGNOSIS CRITERIA** | **AGE, YEARS (M±SD)** | **BMI** | **SS** | **AS** |
| --- | --- | --- | --- | --- | --- | --- | --- | --- |
| Agledahl 2009^(22)^ | 26 | Norway | Low testosterone levels | Serum testosterone ≤11.0 nm/l | 68.9±5.4 | T: 30.6±3.9; C: 29.4±3.9 | Yes and no | NR |
| Amory 2004^(23)^ | 70 | The United States | Low Serum Testosterone | Non-fasting, morning serum total T level below 12.1 nmol/l for 2 days. | 71±4 | T: 28.7±3.6; P: 27.8±3.6 | No | No |
| Arlt 2007^(24)^ | 92 | UK | Low androgen levels | The sulfated DHEA and bioavailable testosterone levels at enrollment were <4.3 μmol/l and <3.6 nmol/l, respectively. | NA | NR | NR | NR |
| Atkinson 2010^(64)^ | 30 | UK | Low to borderline-low testosterone levels | Morning (before 11.00 am) total T ≤ 12 nmol/L or calculated free T ≤ 250 pmol/L. | 72.6 ± 5.7 | T: 28.6 ± 4.2;  P: 28.3 ± 5.3 | NR | NR |
| Aversa 2003^(25)^ | 20 | German | ‘Low-normal’ serum androgen levels | Serum T-values between 10 and 13 nmol/L and serum free testosterone values between 200 and 300 pmol//L | 56 | P:23.7 ± 2.1;  T: 22.9 ± 2.2 | Yes and no | NR |
| Aversa 2010a^(65)^ | 50 | Italy | Late-onset hypogonadism | Total testosterone at or below 11 nmol/L or free testosterone at or below 250 pmol/L | 57±8 | P: 31±6.2 ; Intramuscular TU: 30.2±4.5 | Yes and no | No |
| Aversa 2010b^(66)^ | 52 | Italy | Hypogonadal | Total testosterone serum level 11 nmol/L or calculated free testosterone levels 10 pg/ml | 57±8 | P: 31±6.2 ; Oral TU: 32.5±5.2; Intramascular TU: 30.2±4.5 | NR | No |
| Basaria 2010^(12)^ | 209 | America | Testosterone deficiency | A total serum testosterone level of 100 to 350 ng per deciliter or a free serum testosterone level of less than 50 pg/ml | 74 | T: 29.7±4.1; P: 30.0±4.2 | Yes and no | NR |
| Basurto 2008^(26)^ | 48 | Mexico | Low total serum testosterone | Low normal testosterone level was defined as a value under 1 standard deviation of the mean in normal men aged between 50 to 70 years. | 63.15±7.9 | T: 27.4+3.01; C: 27.2±2.04 | NR | No |
| Behre 2012^(6)^ | 362 | Austria, Finland, Germany, Ireland, Italy, Spain, Sweden, and UK | Testosterone deficiency | (1) Serum total testosterone <15 nmol/L, (2) Bioavailable testosterone <6.68 nmol/L, and (3) An Aging Males’ Symptoms rating scale total score >36 | 61.9 ± 6.6 | T: 28.5 ± 3.3; P: 28.7 ± 3.0 | NR | NR |
| Borst 2014^(27)^ | 60 | America | Hypogonadal | Serum total testosterone≤300 ng/dl or BioT≤70ng/dl. |  | Mean 30.02, Range ≤ 35 | NR | NR |
| Boyanov 2003^(28)^ | 48 | America | Mild androgen deficiency | Have symptoms of andropause or erectile dysfunction, and have serum testosterone levels below the normal range for young adults or in the lower third of this range (total testosterone < 15.1 nmol/L). | 57.5 ± 4.8 | TU: 31.08 ± 4.79;  C: 31.01 ± 4.90 | NR | No |
| Buvat 2011^(29)^ | 173 | France, Italy, Germany, Finland, Spain, Netherlands, UK, USA, etc. (17 European participating centers) | Hypogonadal | Baseline total testosterone levels≤4 ng/mL or bioavailable testosterone ≤1 ng/mL | 58.33± 7.56 | T:27.87±4.19;  P:27.64±3.26 | Yes and no | Yes and no |
| Casaburi 2004^(30)^ | 53 | America | Low testosterone levels | Serum testosterone was 400 ng/dl or less. | 67.7±8.7 | NR | NR | NR |
| Cavallini 2004^(31)^ | 150 | Italy | Androgen decline | Free testosterone lower than 6 pg/mL | 66 years | NR | No | No |
| Christmas2002/Blackman 2002^(32)^ | 74 | America | Late-onset hypogonadism | Serum testosterone levels at least 1standard deviation below the mean for healthy men aged 20 to 39 years. | 72±0.6 | 27.0±0.3 | No | No |
| Clague 1999^(33)^ | 14 | UK | Androgen deficiency | Total testosterone levels < 14 nmol/L. | 68.1±6.6 | NR | NR | NR |
| Del Fabbro 2013^(34)^ | 43 | USA | Hypogonadal | Bioavailable testosterone <70 ng/dL | 63±6 | T: 23±3; P:24±3 | NR | NR |
| Dias 2015^(35)^ | 43 | America | Low testosterone | Testosterone levels < 350 ng/dL | 71 | T:30.12±1.11; P:27.62±1.15 | Yes and no | No |
| Dias 2017^(36)^ | 35 | America | Testosterone deficiency | Fasting morning (7–10 am) total testosterone levels <350 ng/dl | 72 ± 1 | T: 30.1 ± 1.1;  P: 27.6 ± 1.2;  AI: 27.8 ± 1.2 | NR | NR |
| Emmelot-Vonk 2008^(37)^ | 237 | Netherlands | Low testosterone levels | Testosterone level below the 50th percentile of the study population-based testosterone distribution (<13.7 nmol/l). | 67.1±5.0 | T: 27.4±3.8; P: 27.3±3.9 | Yes and no | Yes and no |
| Fennell 2010^(38)^ | 38 | Australia | Androgen deficiency | NR | 52 ± 2 | 29.5 ± 0.8 | NR | NR |
| Frederiksen 2012^(39)^ | 38 | Denmark | Hypogonadal | Bioavailable testosterone < 7.3 nmol/L | Mean 68 | T: 30.2±3.6; P: 30.1±5.0 | NR | NR |
| Fui 2018^(17)^ | 100 | Australia | Obese dieting men with low testosterone levels | BMI >30 kg/m2; two fasting, morning (8–10 am) total testosterone levels ≤12 nmol/L. | NR | T:37.4(34.8,40.6);  P:37.3(34.7,41.6) | NR | NR |
| Gianatti 2014^(67)^ | 88 | Australia | Low testosterone level men with T2DM | Total testosterone level was <12.0 nmol/L. | Mean 62 | T: 31.5 (28.3–35.5); P: 33.4 (31.4–35.4) | NR | NR |
| Hackett 2014^(40)^ | 199 | UK | Hypogonadal men with T2DM | According to the current 200 ISSAM EAU guidelines, and with symptoms of hypogonadism defined by the ageing male symptom | 61.6 ± 9.9 | 32.7 ± 5.8 | NR | NR |
| Hildreth 2013^(41)^ | 167 | America | Low testosterone levels | An average of 2 separate baseline fasting morning total testosterone samples between 200 and 350 ng/dL | 66.5 ± 5.8 | NR | NR | NR |
| Jones 2011^(42)^ | 220 | Belgium, France, Germany, Italy, the Netherlands, Spain, Sweden, and the UK | Hypogonadal men with type 2 diabetes and/or metabolic syndrome | Early morning [08:00-10:00] total testosterone ≤ 11 nmol/L or free testosterone ≤ 255 pmol/L on two occasions ≥ 1week apart | 59.9 ± 9.3 | T: 32.87 ± 6.58;  P: 31.29 ± 5.44 | NR | NR |
| Kenny 2002^(5)^ | 67 | The United States | Low bioavailable testosterone levels | Bioavailable testosterone levels below 4.44 nmol/L. | 76±4 | T: 27±3;  P: 26±3 | No | Yes and no |
| Kenny 2010^(43)^ | 131 | The United States | Men with osteoporosis, physical frailty, and low testosterone | Testosterone levels below 350 ng/dL or bioavailable testosterone levels at least 1.5 standard deviation lower than the young adult mean (95–350 ng/dl for men 40–49yrs). | 77.1±7.6 | 26.9±4.3 | NR | NR |
| Legros 2009^(44)^ | 322 | Austria (1 site), Belgium (3 sites), France (1 site), Germany (2 sites), UK (3 sites), The Netherlands (3 sites), and Switzerland (1 site) | Androgen deficiency | Androgen deficiency (as indicated by a positive score on androgen deficiency in aging males questionnaire) as well as a calculated free morning testosterone measurement of <0.26 nmol/l | 59.5±6.5 | T:(80 mg/day): 27.3±3.4; T(160 mg/day): 27.5±3.5; T (240 mg/day): 26.8±3.5; P: 27.4±3 | NR | NR |
| Liu 2003^(45)^ | 17 | Austria | Testosterone deficiency | Plasma testosterone ≤430 ng/dl on two separate occasions | 67.5 | NR | NR | NR |
| Maggio 2013^(46)^ | 108 | America | Low testosterone | Serum testosterone concentration <475 ng/dL | 71.8 ± 4.9 | T:25.8±3.3 P:24.8±2.4 | NR | NR |
| Marks 2006^(47)^ | 44 | The United States | Late-onset Hypogonadism | A screening testosterone level lower than 300 ng/dL | NR | T: 28.34 (22.7-37.9); P: 29.57 (23.6-37.8) | NR | NR |
| Meier 2004^(48)^ | 37 | America | Partial androgen deficiency | Total testosterone <=15nmol/L | 68.0 ± 6.0 | DHT:26.0±2.7;  P:28.9±3.9 | Yes and no | NR |
| Merza 2006^(49)^ | 39 | The United Kingdom | Borderline hypogonadism | Total testosterone <10 nmol/L or free androgen index <30% | 62.0±9.7 | NR | NR | No |
| Nair 2006^(50)^ | 92 | The United States | Low levels of the sulfated form of DHEA and bioavailable testosterone | A level of bioavailable testosterone that was less than 3.6 nmol /l and a sulfated DHEA level that was less than 1.57 μg/ml. | NR | T: 28.4(25.7-30.3); DHEA: 27.1(24.5-28.9);  P: 27.4(25.9-30.0) | NR | NR |
| Shankar 2010^(57)^ | 274 | The United Kingdom | Low Serum Testosterone | Morning (before 11:00 h) total testosterone of 12 nmol/l or less or calculated free T of 250 pmol/l or less. | 73.7±5.7 | T: 27.9±4.1; P: 27.7±4.0 | NR | NR |
| Shigehara 2011^(51)^ | 52 | Japan | Hypogonadal men with benign prostate hypertrophy | Free testosterone value of ＜11.8 pg/mL | 72±6.5 | NR | NR | NR |
| Shores 2009^(52)^ | 33 | The United States | Hypogonadal older men | Total testosterone levels of ≤ 280 ng/dL | 57.1±5.7 | T: 33.2±4.5; P: 32.9±8.2 | NR | NR |
| Sih 1997^(53)^ | 32 | The United States | Hypogonadism | Hypogonadism was defined as a bioavailable testosterone<60 ng/dL | 65±7 | T: 29.1±5.2; C: 27.3±3.8 | NR | NR |
| Simon D 2001^(54)^ | 18 | France | Low levels of plasma total testosterone | Either plasma total testosterone ≤ 3.4 ng/ml from 1985 to 1987 and ＜4.0 ng/ml from 1992 to 1993 or plasma total testosterone ＜4.0 ng/ml from 1992 to 1993 and ＜ 4.0 ng/ml a few days before inclusion. | 52.8±4.2 | T: 29.9±0.9, DHT:27.8±0.9,  P: 28±1.1 | NR | NR |
| Sinclair 2016^(68)^ | 101 | Australia | Low testosterone | Total testosterone <12nmol/l; or vermeulen-calculated free testosterone <230pmol/l | NR | Median:28.8 Range: [26.0;32.5] | NR | Yes and no |
| Snyder 1999^(55)^ | 108 | The United States | Low serum testosterone and bone mineral density | Serum testosterone concentration at least 1 standard deviation below the mean for healthy young men (<475 ng/dL) and a bone mineral density of the lumbar spine (L2–L4) below the mean for healthy young men (<1.26 g/cm2). | 73.0±5.9 | NR | NR | No |
| Snyder 2017^(18)^ | 211 | America | Low testosterone | A serum testosterone concentration on 2 morning specimens that averaged less than 275 ng/dL. | 72.3±5.9 | 31.2±3.4 | Yes and no | Yes and no |
| Spitzer 2012^(56)^ | 140 | The United States | Erectile dysfunction and low testosterone | Total testosterone levels less than 11.45 nmol/L, or free testosterone levels less than 173.35 pmol/L. | 55.1±8.3 | T: 31.5±6.4; P: 32.7±6 | No | NR |
| Stout 2012^(58)^ | 41 | The United Kingdom | Low testosterone status with chronic heart failure | Low testosterone status was defined as <15 nmol/l, as per local laboratory guidelines, and chronic heart failure as an ejection fraction of <35%. | 68.3±5.3 | T: 28.7±1.8; P: 30.1±3.5 | NR | NR |
| Tan 2003^(59)^ | 10 | The United States | Hypogonadal with Alzheimer’s disease | Total testosterone level < 250 ng/dl. | NR | NR | NR | NR |
| Tan 2013^(60)^ | 120 | Malaysia | Testosterone deficiency syndrome | Total testosterone < 12 nmol/l.  and total Aging Male Symptoms questionnaire scores ≥ 27 | 53.4 ± 7.4 | T: 30.5±5.3; P: 28.3± 4.6 | NR | NR |
| Vaughan 2007^(61)^ | 69 | The United States | Low serum Testosterone | Baseline total testosterone below 350 ng/dl. | 70.8±4.2 | NR | No | No |
| Wang 2013^(62)^ | 186 | China | Low serum testosterone | Serum testosterone < 300 ng/dl. | 68.2 ± 5.2 | 27.9±3.2 | NR | NR |
| Wittert, 2003^(63)^ | 76 | Australia | Androgen Deficiency | At least east 2 symptoms on the Saint Louis University Androgen Deficiency in the Aging Male (ADAM) questionnaire. | 68.5±6 | T: 27±4;  P: 29±5 | Yes and no | NR |

SS: smoking status; AS: alcohol status; T: testosterone; C: control; P: placebo; AI: aromatase inhibitor; DHEA: dehydroepiandrosterone; DHT: double hydrogen testosterone; TU: testosterone undecanoate.

Table 2: Characteristics of interventions

| **STUDY ID** | **METHODS OF INTERVENTIONS** | **DOSAGES/FREQUENCY** | **ROUTE OF ADMINISTRATION** |
| --- | --- | --- | --- |
| Sinclair 2016^(68)^ | Testosterone | Testosterone (1000 mg)  injection time: at 0,6,18,30,42month | Intramuscular |
| Snyder 2017^(18)^ | Testosterone: AndroGel 1%in a pump bottle (AbbVie)  (elemental calcium600g; vitamin D3 400 units; twice a day) | Initial dose: 5 g daily  Later: the dose of testosterone gel was adjusted after each measurement to attempt to keep the concentration within the normal range for young men | Transdermal |
| Dias 2015^(35)^ | Transdermal Testosterone gel + placebo tablet + calcium (1500 mg) and vitamin D (600 IU) daily | 5g TT; daily  6-weeks after randomization, if the TT dose was <500 ng/dL in any subject, the dose was increased by 2.5 g daily and reciprocal ‘sham increase’ with the placebo gel was performed in either the AI-group or the placebo group to maintain the blind. | Transdermal |
| Maggio 2013^(46)^ | Scrotal patch (Testoderm) | 6 mg testosterone/24 h, 60-cm^2^ | Transdermal |
| Wang 2013^(62)^ | TU Andriol Testocaps Calcium (600mg) and vitaminD3 (125 IU) daily | 20mg, per day | Oral |
| Meier 2004^(48)^ | DHT | 70mg/day | Transdermal |
| Christmas2002/Blackman 2002^(32)^ | Human GH + placebo sex steroid | 100mg per two weeks | Intramuscular |
| Arlt 2007^(24)^ | Oral DHEA + transdermal placebo patch | 75mg DHEA; 0mg T | Oral |
| Borst 2014^(27)^ | TE-finasteride | TE:125mg/week Finasteride: 5mg/day | Intramuscular |
| Kenny 2010^(43)^ | Testosterone | 5 mg/d | Other |
| Basurto 2008^(26)^ | Testosterone | 250mg every 21 days | Intramuscular |
| Emmelot-Vonk 2008^(37)^ | Testosterone | 80mg/ twice a day | Oral |
| Nair 2006^(50)^ | Placebo (lactose) tablet + testosterone | Testosterone: 5mg/d | Transdermal |
| Merza 2006^(49)^ | Testosterone | 5mg/d | Transdermal |
| Amory 2004^(23)^ | Testosterone + placebo pill | Testosterone: 200mg/2 weeks; placebo pill: per daily | Intramuscular |
| Snyder 1999^(55)^ | Testosterone patch | 6mg/d | Transdermal |
| Fui 2018^(17)^ | Testosterone | 1000mg: weeks of 0, 6, 16, 26, 36, 46 | Intramuscular |
| Behre 2012^(6)^ | Testosterone | Testosterone gel (5 g, equivalent to 50 mg testosterone) | Transdermal |
| Dias 2017^(36)^ | Transdermal testosterone gel | 5 g/day | Transdermal |
| Fennell 2010^(38)^ | Testosterone | 800 mg once | Subdermal T implants |
| Frederiksen 2012^(39)^ | Testosterone | 5 g gel, containing 50 mg testosterone | Transcutaneous |
| Gianatti 2014^(67)^ | Testosterone undecanoate | 1,000 mg was injected into the upper outer quadrant of the buttock at 0, 6, 18, and 30 weeks | Intramuscular |
| Hackett 2014^(40)^ | Testosterone undecanoate | 1,000 mg at week 0, week 6, week 18, administered by the practice nurse or GP over 5 minutes into the right or left upper outer buttock | Intramuscular |
| Hildreth 2013^(41)^ | Testosterone + progressive resistance training | The T gel were provided in 2.5- or 5.0-g packets. All subjects were initiated on two 2.5-g packets daily (1 T gel and 1 placebo packet in the lower-range T group, and 2 T gel packets in the higher-range T group) | Transdermal |
| Tan 2013^(60)^ | Testosterone | 1000mg, all participants received five injections from the package allocated to them at weeks 0, 6, 18, 30 and 42 after formal enrolment | Intramuscular |
| Jones 2011^(42)^ | Testosterone | 3 g metered-dose 2% testosterone gel (60 mg testosterone, Tostran [also known as Fortigel, Tostrex, Itnogen, Foresta; ProStrakan, Galashiels, Scotland, U.K.]), once daily | Transdermal |
| Kenny 2002^(5)^ | Testosterone + calcium + vitamin D | Two 2.5-mg non-scrotal transdermal patches/d; 5 mg/d total dose. all men received 500 mg calcium and 400 IU of vitamin D supplementation | Transdermal |
| Legros 2009^(44)^ | Testosterone undecanoate | Two capsules of TU in the morning, two placebo capsules in the afternoon, and two placebo capsules in the evening, immediately after meals (total daily dose: 80 mg TU) | Oral |
| Liu 2003^(45)^ | Testosterone esters (250 mg/ml, Sustanon 250, Organon Australia, Sydney, Australia) | The first injection was 2 ml (equal to 500 mg testosterone esters in the treatment group), and the subsequent two injections were 1 ml (250 mg testosterone ester) each. | Intramuscular |
| Agledahl 2009^(22)^ | Testosterone undecanoate | 1000mg | Intramuscular |
| Atkinson 2010^(64)^ | Transdermal testosterone gel | 50 mg daily;  The dose of the gel was adjusted to 75 or 25 mg/day according to serum T at Day 10 and 3 months. Dose adjustment was undertaken if T levels remained outside the target range (18–30 nmol/L); the placebo group therefore received the maximum “dose,” and double blinding was preserved. | Transdermal |
| Aversa 2003^(25)^ | Transdermal testosterone +100 mg Viagra tablet | 5 mg/day | Transdermal |
| Aversa 2010a^(65)^ | Parenteral TU | 1000 mg/every 12 weeks | Intramuscular |
| Aversa 2010b^(66)^ | Oral TU | 2 capsules of 40 mg/twice per day  Equaling a total dose of 160 mg/day | Oral |
| Basaria 2010^(12)^ | Testosterone gel | 10 g/day (containing 100mg testosterone)  if the average of two testosterone measurements was less than 500 ng per deciliter (17.4 nmol per liter) then the dose was increased to 15 g daily; testosterone measurements more than 1000 ng per deciliter (34.7 nmol per liter) then the dose was decreased to 5 g daily. | Transdermal |
| Boyanov 2003^(28)^ | Testosterone undecanoate TU | 120 mg daily | Oral |
| Buvat 2011^(29)^ | 1% hydroalcoholic T gel (50 mg/5 g gel) Tadalafil Once A Day | 50 mg T/5 g gel; Once A Day (if insufficient subjective clinical response after 4 or 8 weeks of combined therapy, increase in T-gel or placebo-gel dose (T gel from 50 mg T/5 g gel up to 100 mg/10 g daily)) | Transdermal |
| Casaburi 2004^(30)^ | Placebo injections +no training | Placebo (sesame oil)/ week | Intramuscular |
| Cavallini 2004^(31)^ | Testosterone undecanoate | 160 mg/day | Oral |
| Clague 1999^(33)^ | Testosterone enanthate | 200 mg/ 2 weeks | Intramuscular |
| Del Fabbro 2013^(34)^ | Testosterone enanthate | 150 or 200 mg | Intramuscular |
| Marks 2006^(47)^ | Testosterone enanthate | 150 mg every 2 weeks | Intramuscular |
| Vaughan 2007^(61)^ | Testosterone + Placebo | 200mg/every 2 weeks | Intramuscular |
| Shigehara 2011^(51)^ | Testosterone enanthate | 250 mg every 4 weeks | Intramuscular |
| Shores 2009^(52)^ | Testosterone | 7.5g/daily | Oral |
| Sih 1997^(53)^ | Testosterone cypionate | 200mg(1ml)/biweekly (every 14-17 days) | Intramuscular |
| Simon D. 2001^(54)^ | Testosterone | 125mg for the first week/daily; adaptation doses aimed at obtaining a trough level of PTT between 4 and 10 ng/ml/between days 10 and 20 | Transdermal |
| Spitzer 2012^(56)^ | Testosterone + sildenafil (2.7±1.4 tablets per week) | 2 tubes(10g) plus 1 tube placebo/daily for 2 weeks. Then, increased the daily testosterone dose to 15 g (3 active tubes) if the mean testosterone level was less than 17.35 nmol/L (500 ng/dL) or decreased it to 5 g (1 active tube plus 2 tubes of placebo gel) if the mean testosterone level was greater than 34.7 nmol/L (1000 ng/dL). | Transdermal |
| Shankar 2010^(57)^ | Testosterone (hydro-alcoholic T gel), The dose of gel was adjusted to 75 or 25 mg/d according to serum T at d 10 and 3 months. | 50 mg/daily | Transdermal |
| Stout 2012^(58)^ | Testosterone + Exercise training | 100 mg(1ml)/once a fortnight | Intramuscular |
| Tan 2003^(59)^ | Testosterone enanthate | 200 mg/every 2 weeks | Intramuscular |
| Christmas2002/Blackman 2002^(32)^ | Testosterone Undercanoate | 80 mg/twice daily | Oral |

DHT: double hydrogen testosterone; DHEA: dehydroepiandrosterone; TU: testosterone undecanoate.
