# Supplementary figures and images for: The effects of testosterone on bone health in males with testosterone deficiency: a systematic review and meta-analysis
Source: BMC Endocr Disord. 2020 Mar 7;20:33. doi: 10.1186/s12902-020-0509-6 (PMC7060639; doi:10.1186/s12902-020-0509-6)

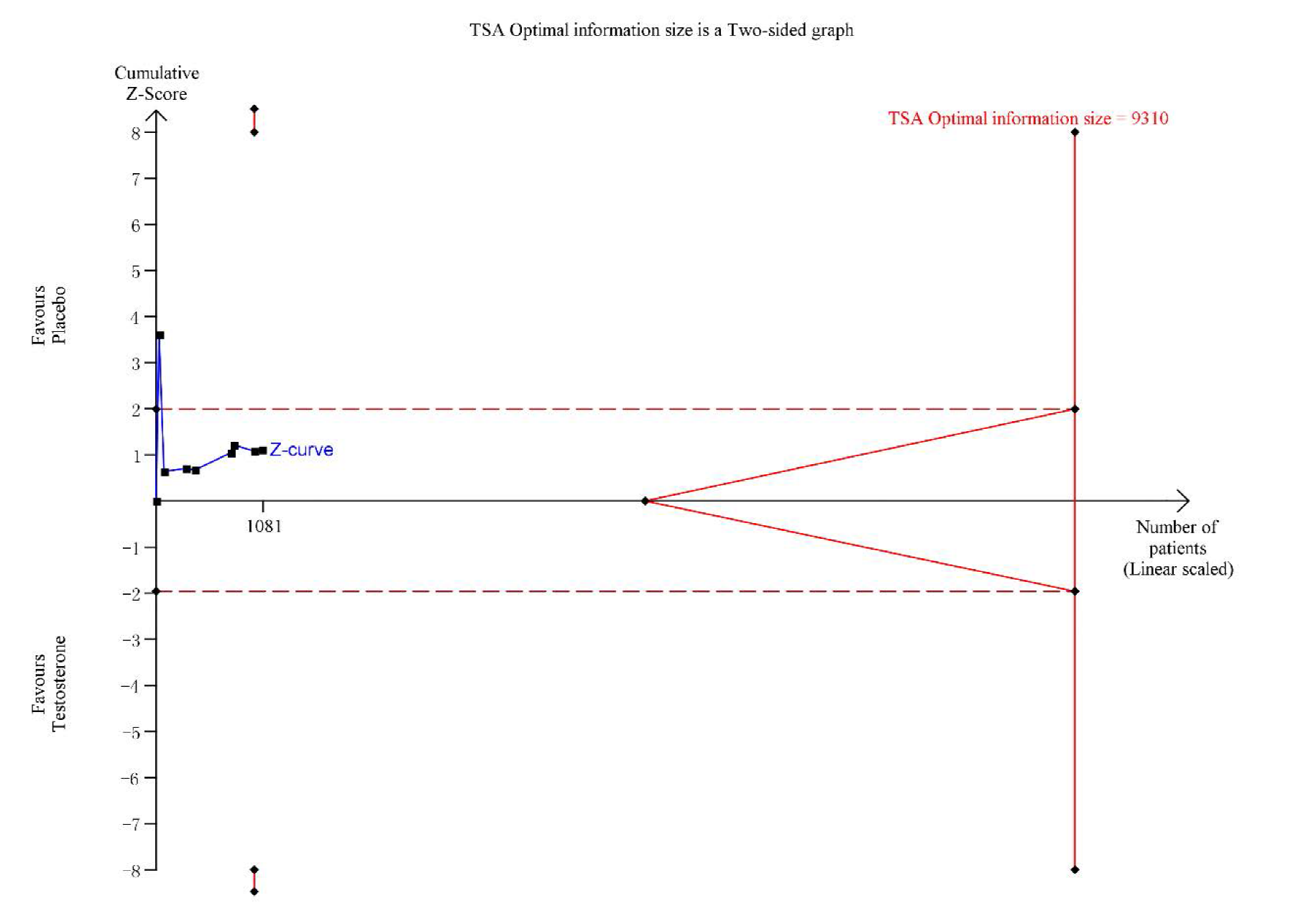

Supplement: Supplementary file 3 — Additional file 3. Trial Sequential Analysis. [file 12902_2020_509_MOESM3_ESM.tif]

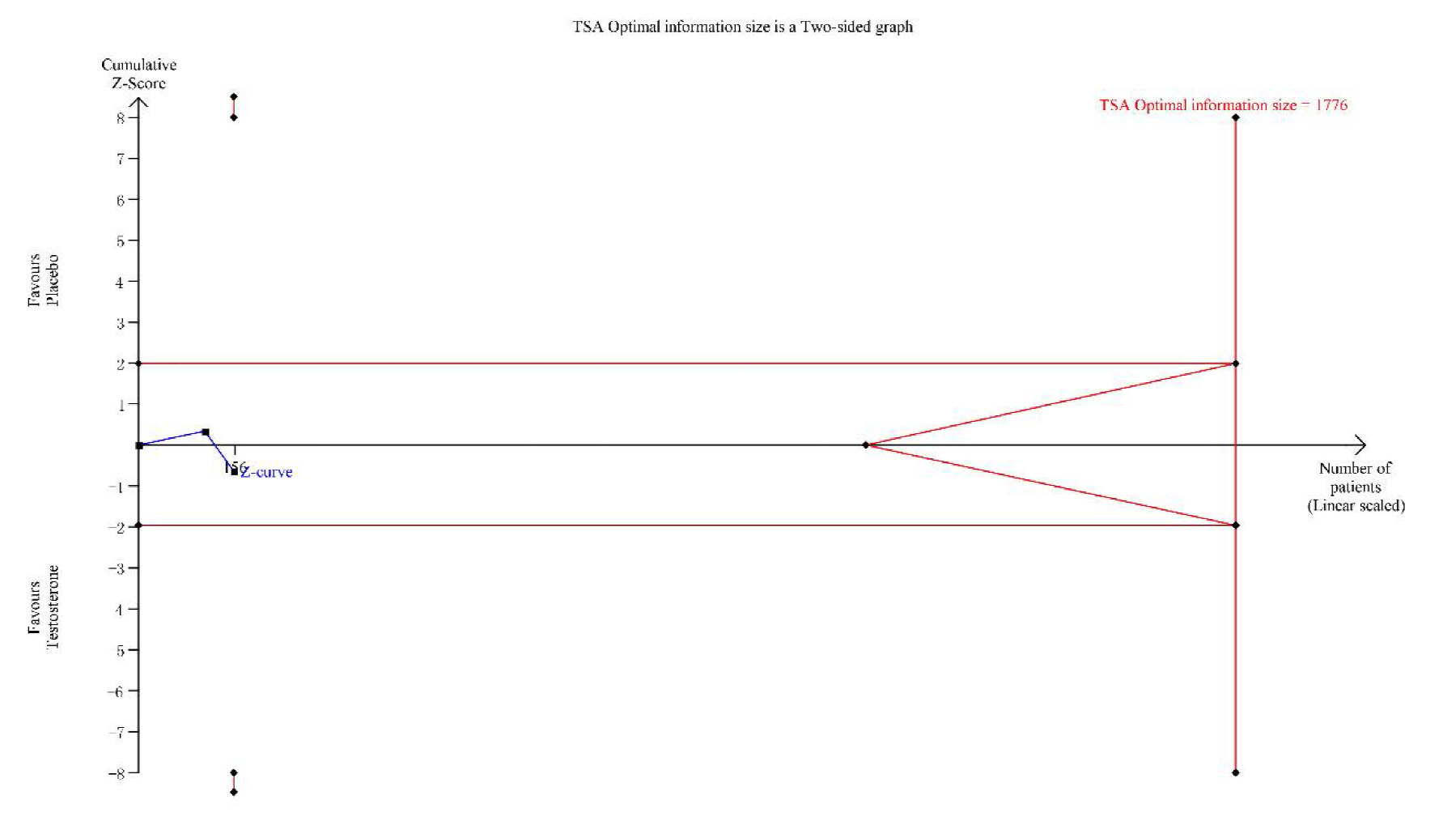

Supplement: Supplementary file 4 — Additional file 4. Testosterone versus placebo: Meta-analysis of quality of life (AMS scale). [file 12902_2020_509_MOESM4_ESM.tif]

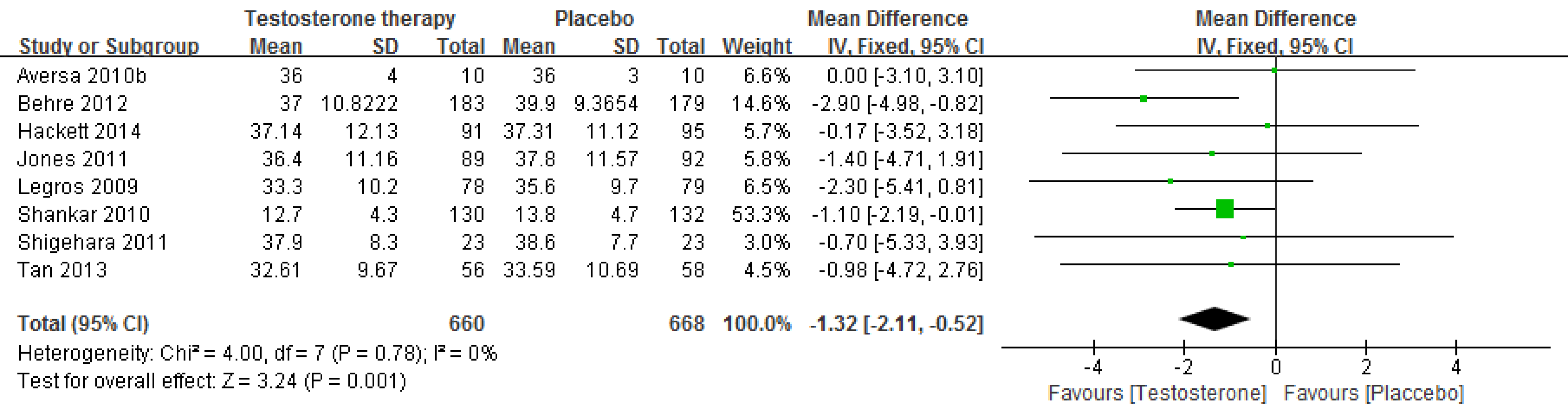

Supplement: Supplementary file 5 — Additional file 5. Testosterone versus placebo: Meta-analysis of sexual function (IIEF-5 scale). [file 12902_2020_509_MOESM5_ESM.tif]

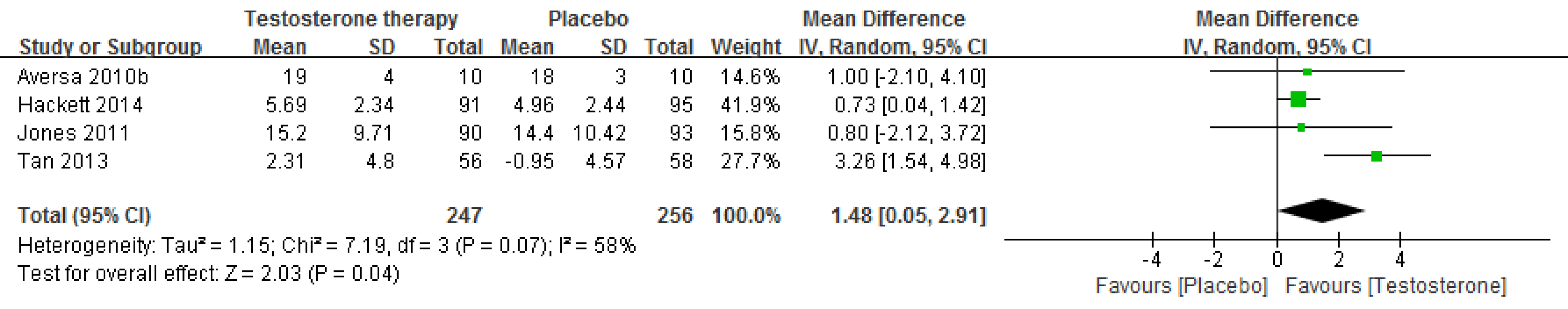

Supplement: Supplementary file 6 — Additional file 6. Testosterone versus placebo: Forest plot of total adverse events. [file 12902_2020_509_MOESM6_ESM.tif]

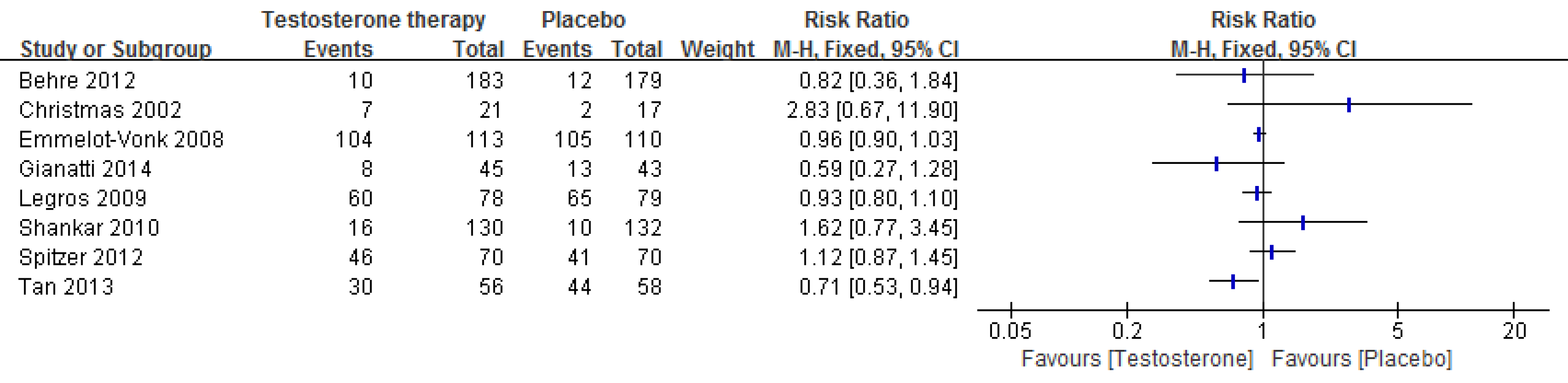

Supplement: Supplementary file 7 — Additional file 7. Testosterone versus placebo: Meta-analysis of serum PSA level. [file 12902_2020_509_MOESM7_ESM.tif]

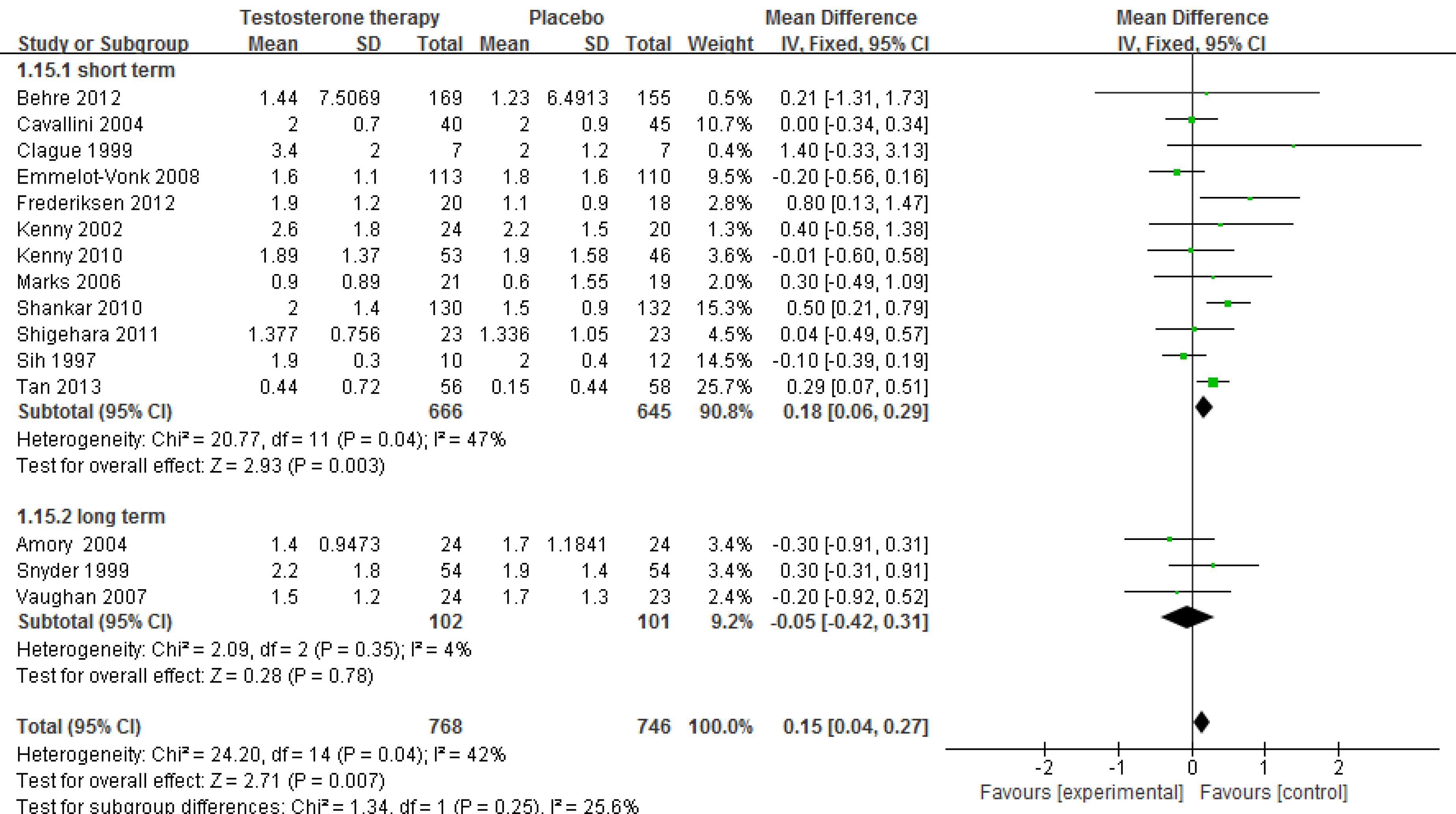

Supplement: Supplementary file 8 — Additional file 8. Testosterone versus placebo: Meta-analysis of risk of PSA increase. [file 12902_2020_509_MOESM8_ESM.tif]

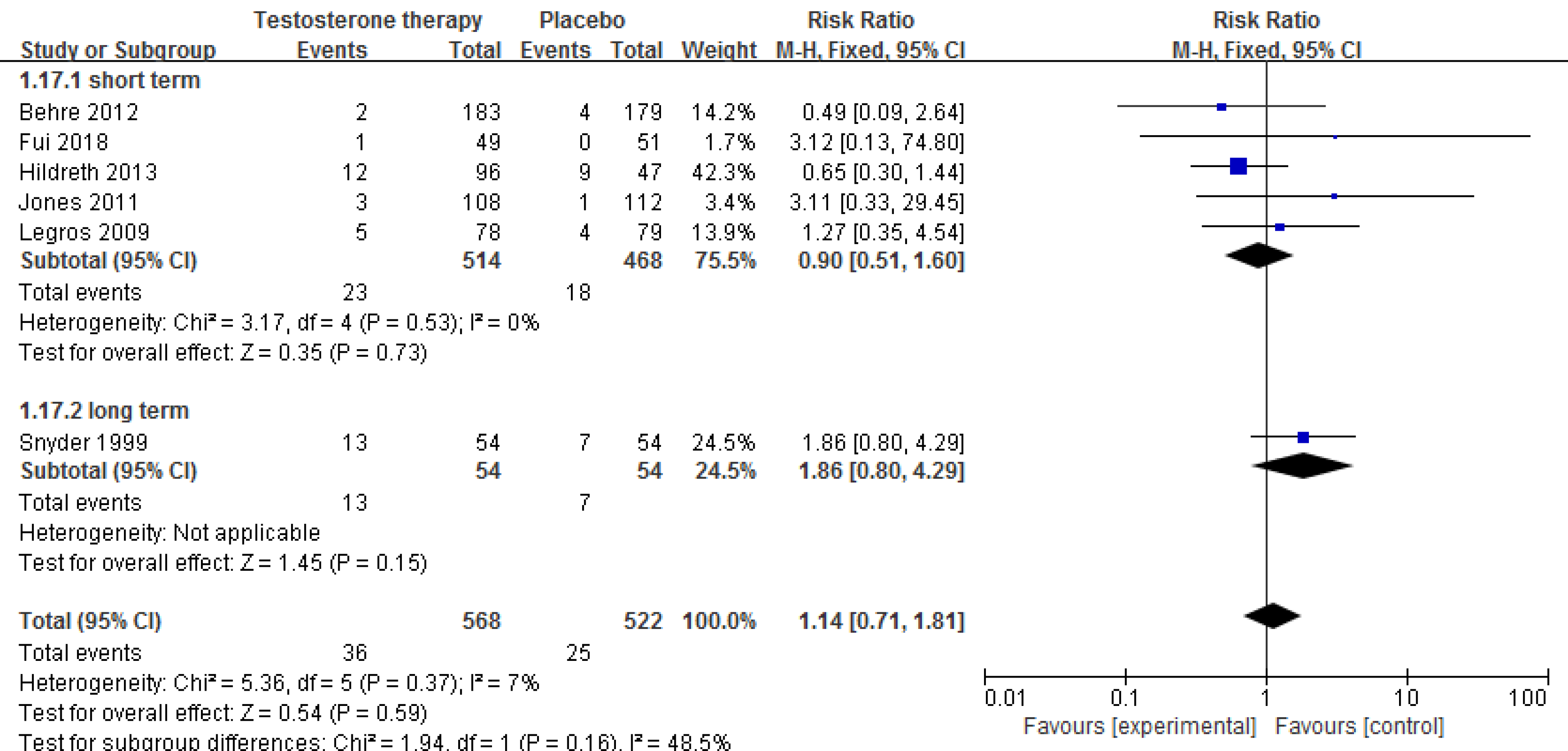

Supplement: Supplementary file 9 — Additional file 9. [file 12902_2020_509_MOESM9_ESM.tif]
