# Supplementary material for: The effects of testosterone on bone health in males with testosterone deficiency: a systematic review and meta-analysis
Source: BMC Endocr Disord. 2020 Mar 7;20:33. doi: 10.1186/s12902-020-0509-6 (PMC7060639; doi:10.1186/s12902-020-0509-6)
Supplement: Supplementary file 10 — Additional file 10. [file 12902_2020_509_MOESM10_ESM.pdf]

National Institute for Health Research

UNIVERSITY of York
Centre for Reviews and Dissemination

# Systematic review

Please complete all mandatory fields below (marked with an asterisk *) and as many of the non-mandatory fields as you can then click *Submit* to submit your registration. You don't need to complete everything in one go, this record will appear in your *My PROSPERO* section of the web site and you can continue to edit it until you are ready to submit. Click *Show help* below or click on the icon
to see guidance on completing each section.
This record cannot be edited because it has been rejected

## 1. * Review title.

Give the working title of the review, for example the one used for obtaining funding. Ideally the title should state succinctly the interventions or exposures being reviewed and the associated health or social problems. Where appropriate, the title should use the PI(E)COS structure to contain information on the Participants, Intervention (or Exposure) and Comparison groups, the Outcomes to be measured and Study designs to be included.

The effects of testosterone on bone health in aging males with testosterone deficiency: a systematic review and meta-analysis

## 2. Original language title.

For reviews in languages other than English, this field should be used to enter the title in the language of the review. This will be displayed together with the English language title.

## 3. * Anticipated or actual start date.

Give the date when the systematic review commenced, or is expected to commence.

20/09/2018

## 4. * Anticipated completion date.

Give the date by which the review is expected to be completed.

30/12/2018

## 5. * Stage of review at time of this submission.

Indicate the stage of progress of the review by ticking the relevant Started and Completed boxes. Additional information may be added in the free text box provided.
Please note: Reviews that have progressed beyond the point of completing data extraction at the time of initial registration are not eligible for inclusion in PROSPERO. Should evidence of incorrect status and/or completion date being supplied at the time of submission come to light, the content of the PROSPERO record will be removed leaving only the title and named contact details and a statement that inaccuracies in the stage of the review date had been identified.
This field should be updated when any amendments are made to a published record and on completion and publication of the review. If this field was pre-populated from the initial screening questions then you are not able to edit it until the record is published.

The review has not yet started: Yes

| Review stage | Started | Completed |
|---|---|---|
| Preliminary searches | No | No |
| Piloting of the study selection process | No | No |
| Formal screening of search results against eligibility criteria | No | No |
| Data extraction | No | No |
| Risk of bias (quality) assessment | No | No |
| Data analysis | No | No |

Provide any other relevant information about the stage of the review here (e.g. Funded proposal, protocol not yet finalised).

## 6. * Named contact.

The named contact acts as the guarantor for the accuracy of the information presented in the register record.

Hongjun Li

## Email salutation (e.g. "Dr Smith" or "Joanne") for correspondence:

Hongjun Li

## 7. * Named contact email.

Give the electronic mail address of the named contact.

hongjun63@163.com

## 8. Named contact address

Give the full postal address for the named contact.

## 9. Named contact phone number.

Give the telephone number for the named contact, including international dialling code.

## 10. * Organisational affiliation of the review.

Full title of the organisational affiliations for this review and website address if available. This field may be completed as 'None' if the review is not affiliated to any organisation.

Department of Urology, Peking Union Medical College Hospital (PUMCH), Chinese Academy of Medical Sciences, Beijing 100730, China

## Organisation web address:

## 11. * Review team members and their organisational affiliations.

Give the personal details and the organisational affiliations of each member of the review team. Affiliation refers to groups or organisations to which review team members belong. **NOTE: email and country are now mandatory fields for each person.**

Zhichao Zhang. Androlofy Center, Department of Urology, Peking University First Hospital, Institute of Urology, Peking University, Beijing, China
Hongjun Li. Urological Department of Peking Union Medical College Hospital (PUMCH), Peking Union Medical College, Chinese Academy of Medical Sciences, Beijing, China
Deying Kang. Department of Evidence based Medicine and Clinical Epidemiology (D-YK), West China Hospital, Sichuan University, Chengdu; and Urological Department of Peking Union Medical College Hospital (PUMCH) (H-JL), Peking Union Medical College, Chinese Academy of Medical Sciences, Beijing, China

## 12. * Funding sources/sponsors.

Give details of the individuals, organizations, groups or other legal entities who take responsibility for initiating, managing, sponsoring and/or financing the review. Include any unique identification numbers assigned to the review by the individuals or bodies listed.

Funding supported by Merch Sharp & Dohme Corp

## Grant number(s)

## 13. * Conflicts of interest.

List any conditions that could lead to actual or perceived undue influence on judgements concerning the main topic investigated in the review.

None

## 14. Collaborators.

Give the name and affiliation of any individuals or organisations who are working on the review but who are not listed as review team members. **NOTE: email and country are now mandatory fields for each person.**

Sai Zhao. Systematic Review Solutions. Ltd
Hangqing Yu. Systematic Review Solutions. Ltd
Zhengnan Cheng. Systematic Review Solutions. Ltd

## 15. * Review question.

State the question(s) to be addressed by the review, clearly and precisely. Review questions may be specific or broad. It may be appropriate to break very broad questions down into a series of related more specific questions. Questions may be framed or refined using PI(E)COS where relevant.

To estimate the effect of testosterone therapy on bone health in aging males with testosterone deficiency.

## 16. * Searches.

State the sources that will be searched. Give the search dates, and any restrictions (e.g. language or publication period). Do NOT enter the full search strategy (it may be provided as a link or attachment.)

We plan to undertake electronic search strategies using MEDLINE via Ovid SP, The Cochrane Library, EMBASE via Ovid SP and PubMed. We plan to search all potentially published relevant RCTs identified in our trial search. There will be no limitations on language or publication period. The searched strategy are developed by an information specialist and are presented in a supplementary file below.

## 17. URL to search strategy.

Give a link to a published pdf/word document detailing either the search strategy or an example of a search strategy for a specific database if available (including the keywords that will be used in the search strategies), or upload your search strategy.Do NOT provide links to your search results.

https://www.crd.york.ac.uk/PROSPEROFILES/109738_STRATEGY_20181011.pdf

Alternatively, upload your search strategy to CRD in pdf format. Please note that by doing so you are consenting to the file being made publicly accessible.

Do not make this file publicly available until the review is complete

## 18. * Condition or domain being studied.

Give a short description of the disease, condition or healthcare domain being studied. This could include health and wellbeing outcomes.

In addition to sexual dysfunction, depressed mood, and fatigue, testosterone deficiency in men can also result in a reduction in bone mineral density. Decreased bone density increases fracture risk that constitute a serious disease burden. A number of randomized controlled trials have demonstrated the beneficial effects of testosterone therapy on BMD. However, the results of these studies have often been inconsistent. This systematic review and meta-analysis is to estimate the effect size of testosterone therapy on bone mineral density and risk of fracture by a combination of results from multiple studies. This meta-analysis will help to resolve the uncertainty about the benefit and safety of testosterone therapy in hypogonadal men with osteoporosis.

## 19. * Participants/population.

Give summary criteria for the participants or populations being studied by the review. The preferred format includes details of both inclusion and exclusion criteria.

Aging male adults (aged greater than or equal to 40 years old), either as inpatients or outpatients, with a diagnosis of testosterone deficiency or late-onset hypogonadism (defined by original studies) will be included in this study.

## 20. * Intervention(s), exposure(s).

Give full and clear descriptions or definitions of the nature of the interventions or the exposures to be reviewed.

Testosterone includes oral TU capsules, Androgel, Fortesta, Axiron gels, Intranasal Natesto, Testim Patches, Androderm Patches, Intramuscular Cypionate, Intramuscular Enanthate, Intramuscular Undecanoate (Aveed), Subdermal Testopel, Sublingual testosterone and Sublingual Buccal testosterone. While Testosterone combined other therapies (such as calcium or vitamin D) will all be included. But patients who received androgen (for instance, finasteride, sildenafil) with testosterone will be excluded.

## 21. * Comparator(s)/control.

Where relevant, give details of the alternatives against which the main subject/topic of the review will be compared (e.g. another intervention or a non-exposed control group). The preferred format includes details of both inclusion and exclusion criteria.

The comparison will be as follows

1) Testosterone therapy alone vs Placebo

2) Testosterone combined other therapy vs other therapy plus placebo.

## 22. * Types of study to be included.

Give details of the types of study (study designs) eligible for inclusion in the review. If there are no restrictions on the types of study design eligible for inclusion, or certain study types are excluded, this should be stated. The preferred format includes details of both inclusion and exclusion criteria.

Any RCT which applies testosterone therapy alone or combined testosterone and other therapies will all be included in this systematic review without limitations of treatment dosage, frequency, and duration.

## 23. Context.

Give summary details of the setting and other relevant characteristics which help define the inclusion or exclusion criteria.

## 24. * Main outcome(s).

Give the pre-specified main (most important) outcomes of the review, including details of how the outcome is defined and measured and when these measurement are made, if these are part of the review inclusion criteria.

Incidence rate of bone mineral density (BMD), including lumbar spine BMD, total hip BMD, or other BMD.

## * Measures of effect

Please specify the effect measure(s) for you main outcome(s) e.g. relative risks, odds ratios, risk difference, and/or 'number needed to treat.

## 25. * Additional outcome(s).

List the pre-specified additional outcomes of the review, with a similar level of detail to that required for main outcomes. Where there are no additional outcomes please state ‘None’ or ‘Not applicable’ as appropriate to the review

Incidence rate of hip fracture, incidence rate of fall, incidence of total fracture, vertebra or non-vertebra fracture, all-cause mortality, incidence of cardiovascular event, quality of life, total cost, sexual function, adverse events, PSA increases and prostate events.

## * Measures of effect

Please specify the effect measure(s) for you additional outcome(s) e.g. relative risks, odds ratios, risk difference, and/or 'number needed to treat.

## 26. * Data extraction (selection and coding).

Describe how studies will be selected for inclusion. State what data will be extracted or obtained. State how this will be done and recorded.

Data from each study will be extracted independently by two separate reviewers using a standardized data extraction form. Any disagreements will be resolved by discussion, with the assistance from a third party if necessary. Where more information relating to a potentially eligible study is lacking, we will contact study authors and request further information. We plan to extract all relevant characteristics of included studies, including

1. General study characteristics (First authors and publication year; geographical setting of the study; trial sponsors)

2. Methods (randomization; participant allocation concealment; blinding of participants and study

investigator; blinding of outcome assessment; measured outcomes; study attrition)

3. Interventions (type and frequency of study drugs; dosages; route of administration)

4.Participants (diagnosis; sample size; age; sex; weight; smoking status; alcohol use; comorbidities; history of fracture, study inclusion and exclusion criteria)

5. Outcomes (measured outcomes; length of follow-up)

6. Results (all dichotomous and continuous results; intention to treat (ITT) and last observation carried forward (LOCF) analyses anticipated)

Where possible, we plan to convert variables that can be reported in different metrics (e.g. times of assessments) to a common metric.

## 27. * Risk of bias (quality) assessment.

Describe the method of assessing risk of bias or quality assessment. State which characteristics of the studies will be assessed and any formal risk of bias tools that will be used.

We will make risk of bias judgement based on methods endorsed by The Cochrane Collaboration (Higgins 2011). We will assess the risk of bias on the domains of patient allocation, blinding, selective reporting, attrition of study participants, as well as any other detected sources of bias that may arise. We will generate a risk of bias table, which provides a graphical overview of all included studies and their rating on a traffic lights-based design, showing studies rated as a 'low' (green), 'unclear' (yellow) or 'high' (red) risk of bias.

## 28. * Strategy for data synthesis.

Provide details of the planned synthesis including a rationale for the methods selected. This **must not be generic text** but should be **specific to your review** and describe how the proposed analysis will be applied to your data.

We will synthesize data using a fixed-effect method for all analyses. An $I^2$ estimate greater than or equal to 50% accompanied by a statistically significant ?$^2$ statistic, will be interpreted as evidence of substantial levels of heterogeneity. Where a substantial heterogeneity is found, we will explore potential sources. If the sources of heterogeneity remains unclear, we will synthesize data using a random-effects model.

1. Dichotomous outcomes: we plan to summarize all dichotomous outcome data using risk ratios (RR) and its 95% confidence interval (CI).

2. Continuous outcomes: we plan to summarize all continuous outcome data using mean differences (MD) and its 95% confidence interval (CI). Where we identify skew data from studies of less than 200 participants, we plan to diagnose skewed data from means and standard deviations of our pre-determined outcomes. We will assess whether, with positive measurements, where “the mean is smaller than twice the standard deviation the data are likely to be skewed”.

## 29. * Analysis of subgroups or subsets.

State any planned investigation of ‘subgroups’. Be clear and specific about which type of study or

participant will be included in each group or covariate investigated. State the planned analytic approach.

Subgroup population

The details of the subgroup are shown as below:

• Age

• BMI (obesity versus non obesity )

• Smoking: yes vs no

• Alcoholic: yes vs no

• Comorbidity: with diabetes vs without diabetes

• Comorbidity: with psychotic disorder (anxiety, depression) vs without psychotic disorder

• Comorbidity: with central nervous system disease (cognitive impairment or dementia/Alzheimer's disease) versus without central nervous system disease

• Comorbidity: with cardiovascular disease (hypertension or other cardiac disease) vs without cardiovascular disease

• Comorbidity: with prostate disease vs without prostate disease

• Other subgroup analysis will be considered if data is available (history of fracture, TT)

1) Treatment duration

2) Dosage of testosterone

We plan to use GRADE approach to assess the quality of evidence body of the following outcomes: total BMD, risk of fracture, risk of fall, mortality, risk of cardiovascular events, quality of life and sexual function.

We plan to conduct trial sequential analysis (TSA) for primary outcomes to test the robustness of the synthetic results. The required information size (RIS) will be calculated based on empirical mean difference and variance with a two side alpha of 0.05 and beta of 0.20.

## 30. * Type and method of review.

Select the type of review and the review method from the lists below. Select the health area(s) of interest for your review.

### Type of review

Cost effectiveness
No

Diagnostic
No

Epidemiologic
No

Individual patient data (IPD) meta-analysis
No

Intervention
Yes

Meta-analysis
Yes

Methodology
No

Narrative synthesis

No

Network meta-analysis
No

Pre-clinical
No

Prevention
No

Prognostic
No

Prospective meta-analysis (PMA)
No

Review of reviews
No

Service delivery
No

Synthesis of qualitative studies
No

Systematic review
Yes

Other
No

## Health area of the review

Alcohol/substance misuse/abuse
No

Blood and immune system
No

Cancer
No

Cardiovascular
No

Care of the elderly
No

Child health
No

Complementary therapies
No

Crime and justice
No

Dental
No

Digestive system
No

Ear, nose and throat
No

Education
No

Endocrine and metabolic disorders
Yes

Eye disorders

No

General interest
No

Genetics
No

Health inequalities/health equity
No

Infections and infestations
No

International development
No

Mental health and behavioural conditions
No

Musculoskeletal
No

Neurological
No

Nursing
No

Obstetrics and gynaecology
No

Oral health
No

Palliative care
No

Perioperative care
No

Physiotherapy
No

Pregnancy and childbirth
No

Public health (including social determinants of health)
No

Rehabilitation
No

Respiratory disorders
No

Service delivery
No

Skin disorders
No

Social care
No

Surgery
No

Tropical Medicine
No

Urological
Yes

Wounds, injuries and accidents
No

Violence and abuse
No

## 31. Language.

Select each language individually to add it to the list below, use the bin icon to remove any added in error.
English

There is an English language summary.

## 32. * Country.

Select the country in which the review is being carried out from the drop down list. For multi-national collaborations select all the countries involved.

China

## 33. Other registration details.

Give the name of any organisation where the systematic review title or protocol is registered (such as with The Campbell Collaboration, or The Joanna Briggs Institute) together with any unique identification number assigned. (N.B. Registration details for Cochrane protocols will be automatically entered). If extracted data will be stored and made available through a repository such as the Systematic Review Data Repository (SRDR), details and a link should be included here. If none, leave blank.

## 34. Reference and/or URL for published protocol.

Give the citation and link for the published protocol, if there is one

Give the link to the published protocol.

Alternatively, upload your published protocol to CRD in pdf format. Please note that by doing so you are consenting to the file being made publicly accessible.

No I do not make this file publicly available until the review is complete

Please note that the information required in the PROSPERO registration form must be completed in full even if access to a protocol is given.

## 35. Dissemination plans.

Give brief details of plans for communicating essential messages from the review to the appropriate audiences.

## Do you intend to publish the review on completion?

Yes

## 36. Keywords.

Give words or phrases that best describe the review. Separate keywords with a semicolon or new line. Keywords will help users find the review in the Register (the words do not appear in the public record but are included in searches). Be as specific and precise as possible. Avoid acronyms and abbreviations unless these are in wide use.

## 37. Details of any existing review of the same topic by the same authors.

Give details of earlier versions of the systematic review if an update of an existing review is being registered, including full bibliographic reference if possible.

## 38. * Current review status.

Review status should be updated when the review is completed and when it is published. For newregistrations the review must be Ongoing.
Please provide anticipated publication date

Review_Ongoing

## 39. Any additional information.

Provide any other information the review team feel is relevant to the registration of the review.

## 40. Details of final report/publication(s).

This field should be left empty until details of the completed review are available.

Give the link to the published review.
